# CircMAST1 inhibits cervical cancer progression by hindering the N4-acetylcytidine modification of YAP mRNA

**DOI:** 10.1186/s11658-024-00540-6

**Published:** 2024-02-08

**Authors:** Chunyu Zhang, Li Yuan, Qiaojian Zou, Caixia Shao, Yan Jia, Jiaying Li, Yan Liao, Xueyuan Zhao, Weijia Wen, Xu Jing, Guofen Yang, Wei Wang, Hongye Jiang, Shuzhong Yao

**Affiliations:** 1 Department of Obstetrics and Gynecology, The First Affiliated Hospital, Sun Yat-sen University, Guangzhou, China; 2 Guangdong Provincial Clinical Research Center for Obstetrical and Gynecological Diseases, Guangzhou, China; 3 Department of Microbiology, Tumor and Cell Biology, Karolinska Institute, Stockholm, Sweden

**Keywords:** Cervical cancer, Circular RNA circMAST1, N4-acetylcytidine, NAT10, YAP

## Abstract

**Background:**

Cervical cancer (CCa) is the fourth most common cancer among females, with high incidence and mortality rates. Circular RNAs (circRNAs) are key regulators of various biological processes in cancer. However, the biological role of circRNAs in cervical cancer (CCa) remains largely unknown. This study aimed to elucidate the role of circMAST1 in CCa.

**Methods:**

CircRNAs related to CCa progression were identified via a circRNA microarray. The relationship between circMAST1 levels and clinicopathological features of CCa was evaluated using the clinical specimens and data of 131 patients with CCa. In vivo and in vitro experiments, including xenograft animal models, cell proliferation assay, transwell assay, RNA pull-down assay, whole-transcriptome sequencing, RIP assay, and RNA-FISH, were performed to investigate the effects of circMAST1 on the malignant behavior of CCa.

**Results:**

CircMAST1 was significantly downregulated in CCa tissues, and low expression of CircMAST1 was correlated with a poor prognosis. Moreover, our results demonstrated that circMAST1 inhibited tumor growth and lymph node metastasis of CCa. Mechanistically, circMAST1 competitively sequestered N-acetyltransferase 10 (NAT10) and hindered Yes-associated protein (YAP) mRNA ac4C modification to promote its degradation and inhibit tumor progression in CCa.

**Conclusions:**

CircMAST1 plays a major suppressive role in the tumor growth and metastasis of CCa. In particular, circMAST1 can serve as a potential biomarker and novel target for CCa.

**Supplementary Information:**

The online version contains supplementary material available at 10.1186/s11658-024-00540-6.

## Introduction

Cervical cancer (CCa) is the fourth most common cancer among females worldwide . Recently, we have witnessed remarkable improvements in the management of CCa due to the introduction of formalized screening strategies and Human Papillomavirus (HPV) vaccination, although CCa still has high incidence and mortality rates in low- and middle-income countries. Surgery and radiotherapy are currently the main strategies for CCa patients; however, they are not ideal for recurrent or advanced CCa [2, 3]. Therefore, elucidating the precise mechanisms CCa progression is pivotal to developing more specific treatment options.

Circular RNAs (circRNAs) are covalently closed transcripts that generated from the back-splicing of pre-mRNAs [4, 5]. in comparison with their linear counterparts, circRNAs have higher stability and resistance to RNase R treatment [6, 7]. Previous reports have confirmed the tissue-specific expression profiles of circRNAs. Accumulating evidence indicates that circRNAs may be involved in the progression of various cancers, such as breast cancer[8], lung cancer[9], cervical cancer [10], etc.

In our previous study, we found that circular RNA hsa_circ_0043280 can inhibit lymph node metastasis of cervical cancer via the miR-203a-3p/PAQR3 axis [11]. Circular RNAs can also function as protein scaffolds regulating gene expression in CCa progression [12, 13]. Our previous study revealed that circVPRBP can promote RACK1 degradation and prevent nodal metastasis in CCa by binding to RACK1 and shield an S122 O-GlcNAcylation site [14]. However, the mechanisms by which circRNAs modulate CCa progression remain largely unknown and need to be further investigated.

RNA undergoes distinct chemical modifications after transcription, leading to expanded regulatory role in RNA function [15–18]. N4-acetylcytidine (ac4C) modification is a kind of conserved RNA modification. Previous studies demonstrated that ac4C is present mainly in 18S rRNA and tRNA [19, 20]. Recent studies reported that ac4C could also occur in mRNA and participate in regulating mRNA stability and translation efficiency in humans [19]. Up to now, N-acetyltransferase 10 (NAT10) is the only reported enzyme regulating ac4C deposition [19–21]. NAT10 promotes N4-acetylation and stabilization of FSP1 mRNA, resulting in colon cancer progression by inhibiting ferroptosis [22]. However, whether ac4C modification plays a biological role in tumor growth and metastasis of CCa and specific mechanisms is currently known. Moreover, non-coding RNAs may regulate gene expression and disease progression by affecting mRNA ac4C modification. LINC00623 bound to NAT10 and blocked its ubiquitination-dependent degradation, thereby remodeling ac4C modification of mRNAs in pancreatic cancer [23]. However, whether circRNA can regulate ac4C acetylation of mRNA remains largely unknown.

In the present study, we identified a novel inhibitory circRNA based on the circRNA microarray data of CCa tissues. We confirmed that circMAST1 inhibited tumor growth and metastasis in CCa. First, we demonstrated that circMAST1 was downregulated in CCa tissues compared with normal cervical tissues, and downregulation of circMAST1 expression was significantly correlated with a poor prognosis. Then, we confirmed that circMAST1 overexpression markedly inhibited the proliferation, migration, invasion, and metastasis of CCa cells in vitro and in vivo. Mechanistically, our study demonstrated that circMAST1 functions via its 160–230 sequence, which interacts with NAT10. We also confirmed that circMAST1 sequestered NAT10 and inhibited NAT10-mediated ac4C modification of YAP mRNA, reducing YAP mRNA stability and suppressing tumor growth and metastasis. Therefore, circMAST1 can serve as a potential prognostic biomarker and therapeutic target for CCa patients.

## Materials and methods

### Clinical specimens

We collected the normal cervical tissues and CCa tissues from patients who underwent surgery at the First Affiliated Hospital of Sun Yat-sen University (Guangzhou, China) from 2010 to 2017. None of the patients that we enrolled had radiotherapy or chemotherapy before surgery. Patients at stages Ia2 to IIa2 were enrolled and the regular follow-up was performed. We collected CCa tissues from patients who had radical hysterectomy and lymphadenectomy, while we obtained normal cervical tissues from patients who had hysterectomy because of non-malignant conditions. Once surgical removal, obtained normal cervical tissues and CCa tissues and were stored at -80 ℃ until RNA and protein extraction. This study was conducted in accordance with the Declaration of Helsinki and was approved by the Ethical Review Committee of the First Affiliated Hospital of Sun Yat-sen University.

### Cell culture

A normal cervical cell line (H8) and human CCa cell lines, SiHa (HPV16 positive), HeLa (HPV18 positive), HeLa229 (HPV18 positive), ME180 (HPV positive), MS751 (HPV45 positive), and Caski (HPV16 positive) were purchased from the American Type Culture Collection (ATCC, USA). H8, HeLa, and SiHa cells were cultured in DMEM (Gibco, USA), HeLa229 and Caski cells were cultured in RPMI1640 (Gibco, USA), and MS751 cells were cultured in MEM (Gibco, USA). All media were supplemented with 1% penicillin/streptomycin (Gibco, China) and 10% fetal bovine serum (FBS) (Gibco, USA). All those cell lines have been assessed by short tandem repeat (STR) genotyping for authenticity, and were cultured in a humid atmosphere with 5% CO^2^ at 37℃. Mycoplasma contamination was regularly screened using the e-Myco Mycoplasma PCR Detection Kit.

### Mice models

Female BALB/c nude mice (4–6 weeks, 18–20 g) were used in this study which were obtained from the Experimental Animal Center of Sun Yat-sen University. All animal procedures evolved in this study were approved by the Sun Yat-sen University Animal Care Committee and raised in SPF conditions. 5×10^6^/150 µL CCa cells were injected into the shoulder of female nude mice to evaluate cervical cancer cells growth in vivo under different conditions. After injection, we monitored and recorded the xenograft tumors every week. 30 days later, all of the mice that have been transplanted with CCa cells were sacrificed to evaluate tumor volume and weight of xenograft tumors. To evaluate the lymph node metastasis capacity, a nude mice model of xenograft lymph node metastasis were used in this study. In brief, 3×10^6^/50µl CCa cells under different conditions were injected into the foot pad of female nude mice. In the indicated time, we removed the footpad tumor and lymph nodes, and then measured their volume to evaluate the influence of circMAST1 on CCa progression. The removed primary tumor and lymph nodes were embedded in paraffin for subsequent immunofluorescence and immunohistochemistry staining. A simple random grouping principle was used in this study to allocate mice into different groups. The volume of primary tumor and lymph nodes was calculated as the following formula: Volume (mm^3^) = 0.52×(length [mm])×(width [mm])^2^.

### RNA and genomic DNA(gDNA) extraction

We used the SteadyPure Universal RNA Extraction Kit (ACCURATE BIOTECHNOLOGY (Hunan) CO., LTD, Changsha, China) to extract the total RNA from cells or tissues following the manufacturer’s instructions. A Fastpure Cell/Tissue DNA Isolation Mini Kit (Vazyme, China) was used to extract the genomic DNA.

### Cytoplasmic and nuclear RNA isolation

A PARIS Kit (Ambion, Life Technologies, USA) to isolate the nuclear and cytoplasmic fractions. Then, the extracted RNA were analyzed by qRT-PCR to clarify the subcellular localization of circMAST1. GAPDH, circ0043280and U6 were used as positive control of cytoplasmic transcript and nuclear transcript respectively.

### Western blotting, immunohistochemistry (IHC), H&E staining, qRT-PCR, RT-PCR, and gel electrophoresis

Western blotting, IHC, H&E staining, qRT-PCR, RT-PCR, and gel electrophoresis were conducted as described previously [24, 25]. Primers used in this study were synthesized by GENEWIZ (Suzhou, China). Primer sequences were provided in the Additional file 1: Table S1. The primary antibodies used were provided in the Additional file 1: Table S1. Immunostained results were captured and evaluated under an optical microscope (Leica, DMI6B, Germany).

### RNA extraction and actinomycin D treatment

We used the SteadyPure Universal RNA Extraction Kit (ACCURATE BIOTECHNOLOGY (Hunan) CO., LTD, Changsha, China) to extract the total RNA from cervical cells following the manufacturer’s instructions. HeLa and SiHa cells were pre-treated with of actinomycin D at a dose of 2 µg/mL (Sigma, USA) to inhibit the novel transcription in the indicated time point. Next, we collected and extracted the total RNA from these cells performed qRT-PCR to evaluate the stability of YAP mRNA.

### Plasmid construction and lentivirus production

The full length of wild type circMAST1 cDNA was cloned into the lentiviral pLC5-Puro vector to ectopically overexpress circMAST1. The mutant CircMAST1 plasmid was synthesized by GENEWIZ (Suzhou, China). The full length of NAT10 cDNA was cloned into the lentiviral pSin-EF1-Puro vector to overexpress NAT10. To construct shRNA-expression vectors, we synthesized oligos for shRNAs and cloned them into pLKO.1-puro plasmids. In this study, X-tremeGENE HP DNA Transfection Reagent (Roche, Germany) was used to transfer the corresponding plasmids into indicated cells following the manufacturer’s instructions. pMD2.G vector (2.5 µg), psPAX2 vector (7.5 µg), and an expression vector of interest (10 µg) were conferred into LentiX-293T cells with 70–80% confluence cultured in a 10-cm dish to produce lentiviral particles. 48 and 72 h later, we collected the supernatant of LentiX-293T cells and filtered through 0.45 µm Filter Unit. The enriched virus precipitation by Lenti-Concentin Virus Precipitation Solution (ExCell Bio) was suspended with PBS and frozen at −80 ℃, or infected cells immediately.

### Cell proliferation assay and transwell assay

Colony formation assays and CCK8 were performed to compare the ability of cells proliferation in vitro[11]. A 24-well plate Transwell system was used to evaluate the ability of cell migration and invasion. In brief, Chambers (8 µm pore size, Corning) were used with or without pre-coated Matrigel (BD Science, USA). Then, we seeded 5 × 10^4^ cervical cancer cells incubated without FBS into the upper chambers, whereas the lower chambers were supplemented with 10% FBS complete culture medium. After 24 h, we fixed the cells with 4% paraformaldehyde for 15 min. Then, 0.1% crystal violet was used to stained migrated or invaded cells. Next, we captured and counted the migrated and invaded cervical cancer cells under a microscope.

### RNA sequencing

Trizol reagent (Invitrogen) were used to extract the total RNA from samples and stored at −80℃. Agilent 2200 were used to check the extracted RNA quality, while RNA sample with RNA integrity number (RIN) > 7.0 was used for subsequent cDNA library construction. cDNA libraries were constructed for each RNA sample using the TruSeq Stranded mRNA Library Prep Kit (Illumina, Inc.) following manufacturer’s instructions. Poly-A-containing mRNA was purified from 1 ug total RNA using oligo(dT) magnetic beads and fragmented into 200–600 bp using divalent cations (at 85℃ for 6 min). Cleaved RNA fragments were used for first- and second-strand complementary DNA (cDNA) synthesis. dUTP mix was used for second-strand cDNA synthesis. cDNA fragments underwent end repair, A-tail was added, and then cDNA fragments were ligated with indexed adapters. The ligated cDNA products were purified and treated with uracil DNA glycosylase to remove the second-strand cDNA. Purified first-strand cDNA was enriched by PCR to construct cDNA libraries. The libraries were quality-controlled with Agilent 2200 and sequenced by NovaSeq 6000 on a 150 bp paired-end run.

### RNA immunoprecipitation (RIP)

A Magna RIP RNA-Binding Protein Immunoprecipitation Kit (Millipore, USA)used in the ac4C-RIP experiments to evaluate ac4C modification on YAP mRNA[11]. First, 1% formaldehyde was pre-cooling and used to cross-linked 2 × 10^7^ CCa cells cultured in the 15cm dish. Then, we collected the cell extracts and incubated with an anti-ac4C or anti-NAT10 antibody at 4℃ overnight. The next day, protein A/G Dynabeads were used to clear the RNA-protein complexes, and then the RNA molecules were then extracted from RNA-protein complexes and analyzed by qRT-PCR.

### RNA pull-down assay and mass spectrometry analysis

We used a Magnetic RNA-Protein Pull-Down Kit (Cat# 20164, Thermo Fisher Scientific) to perform RNA pull-down. We amplified different truncated versions of circMAST1 using the T7 promoter, and TranscriptAid T7 high-yield transcription kit (Cat# K0441, Thermo Fisher Scientific) was used to transcribe RNA in vitro according to the instructions. Then, biotin labeling was performed using the RNA 3 'End biotinylation kit (Cat# 20160, Thermo Fisher Scientific), followed by incubation of 50 pmol of 3' -biotinated transcribed RNA with streptavidin magnetic beads, and then incubation with cell lysate. Antisense RNAs of circMAST1 were used as the negative control. Retrieved proteins were used for subsequent mass spectrometry (MS) (Fitgene Biotech, Guangzhou, China) and Western blotting. MS analysis was conducted using a Q exactive hybrid quadrupole-orbitrap mass spectrometer (ThermoFisher Scientific). MS data have been provided in the Additional file 1: Table S3. Protein identification was performed using MASCOT software by searching Uniprot_Aedis Aegypti.

### Fluorescence in situ hybridization (FISH)

FITC-labelled circMAST1 probe was designed and synthesized by Geneseed (Guangzhou, China). Following manufacturer's instructions, a Fluorescent in Situ Hybridization Kit (RiboBio, Guangzhou, China) were used for Hybridizations. All images were captured using a laser scanning confocal microscope (TCS SP2 AOBS). The probe sequences are shown in the Additional file 1: Table S1.

### Statistical analysis

We used SPSS version 21.0 and GraphPad Prism version 9.0 to conduct statistical analysis. The differences between the two groups were analyzed by using unpaired student’s t-test, and differences between more than two groups were assessed by using one-way analysis of variance (ANOVA). We used Kaplan-Meier method to analyze overall survival, and the log-rank test was used for statistical significance. We used The *χ*^2^ test and Fisher’s exact test to analyze the relationships between circMAST1 expression and clinicopathologic characteristics. Data in this study are presented as mean ± standard deviation (SD) of at least three independent experiments. *P* value < 0.05 was considered statistically significant.

## Results

### Identification and characteristics of circMAST1

**Fig. 1:**
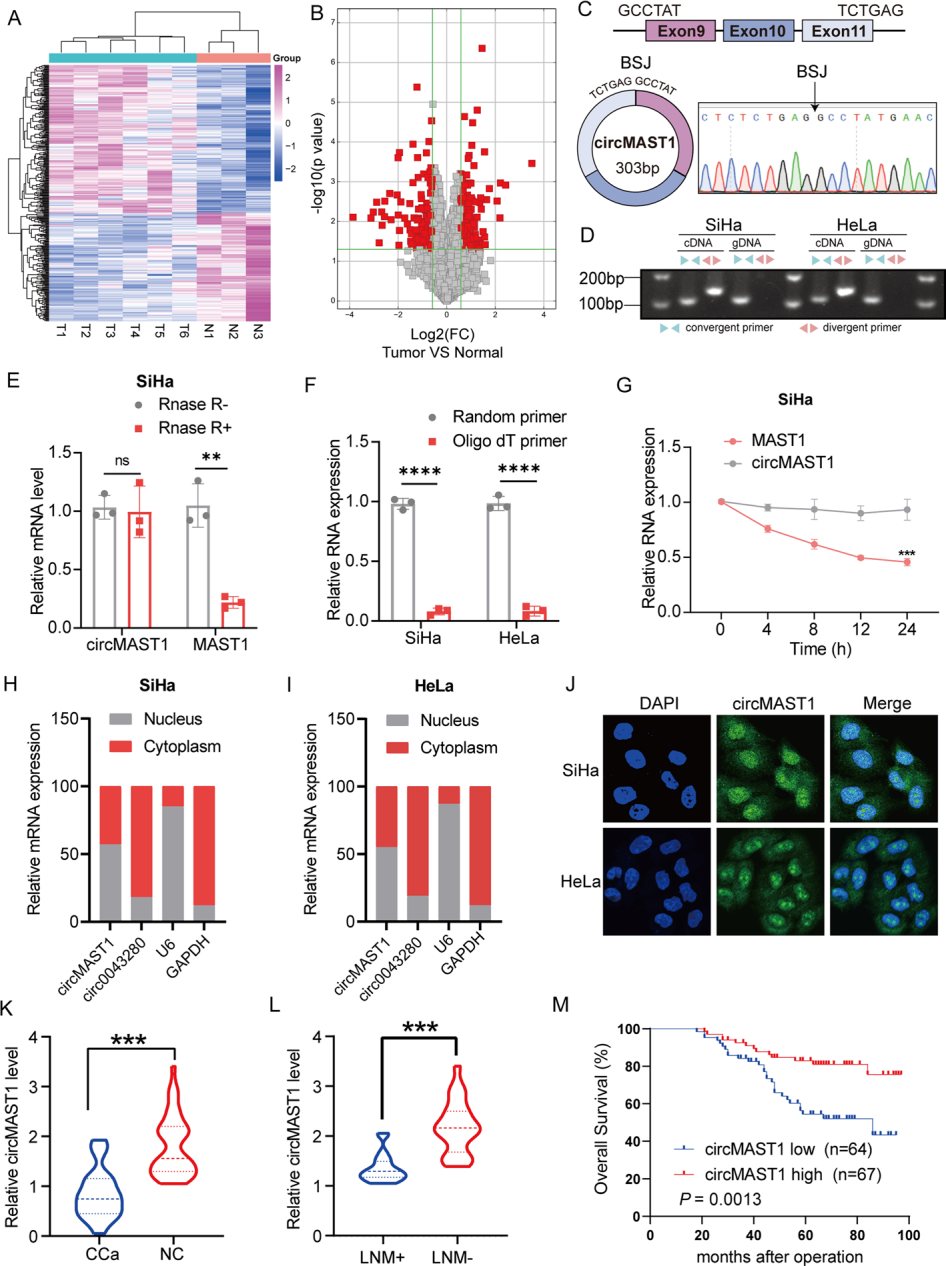
Identification and characterization of circMAST1 in CCa cells and tissues. **A**,** B** The differentially expressed circRNAs in six CCa tissues and three normal cervix tissues were shown as the heatmap matrix and volcano plot. **C** The genomic loci of the circMAST1 gene. circMAST1 is synthesized at the MAST1 gene locus containing exons 9 to 11. The back-splice junction of circMAST1 was identified by Sanger sequencing. **D** PCR analysis for circMAST1 in cDNA and genomic DNA (gDNA) in SiHa and HeLa cells. **E** The qRT-PCR analysis for the expression of circMAST1 and MAST1 mRNA after treatment with RNase R in SiHa cells. **F** The qRT-PCR analysis of circMAST1 using random primers and oligo dT primers, respectively, in reverse transcription experiments. circMAST1 was notably absent in polyA-enriched samples. **G** The qRT-PCR analysis for the expression of circMAST1 and MAST1 mRNA after treatment with actinomycin D at the indicated time points in SiHa cells. **H**,** I** Cytoplasmic and nuclear mRNA fractionation experiments showing that circMAST1 is localized mainly in the nucleus of SiHa and HeLa cells. GAPDH and circ0043280 were applied as positive controls in the cytoplasm, U6 was used as positive control of nucleus. **J** RNA fluorescent in situ hybridization for circMAST1 in SiHa and HeLa cells; the junction probe was complementary to the back-splice junction sequence of circMAST1. Nuclei were stained with DAPI. Scale bar, 20 µm. **K** Expression levels of circMAST1 in CCa tissues in comparison with matched normal tissues were measured using qRT-PCR. **L** Expression levels of circMAST1 in CCa tissues with or without lymph node metastasis were measured using qRT-PCR. **M** Kaplan–Meier survival curves showed poor overall survival with low expression levels of circMAST1. Each experiment was performed at least three times independently. **P < 0.01. ***P < 0.001. ****P < 0.0001

To investigate the biological functions of circular RNAs in CCa progression, we performed a circRNA microarray with 9 cervical samples, including 6 cervical cancer tissues and 3 normal cervical tissues. The results of the microarray showed 172 circRNAs that were significantly downregulated in cervical cancer tissues (the threshold values were |FC (fold change)| ≥ 1 and p < 0.05) (Fig. 1A, B, Additional file 2). Since circMAST1 (hsa_circ_0049613) was the most significantly downregulated circRNA in cervical cancer tissues, we chose circMAST1 for further studies. We found that hsa_circ_0049613 was associated with exons 9 to 11 of the MAST1 gene and possessed 303 nucleotides in length. Next, we designed divergent primers to amplify the back-spliced junction of circMAST1 and sequenced by Sanger sequencing (Fig. 1C); results showed that the amplified sequence was consistent with that available at the circbase database (http://www.circbase.org/). Then, we used convergent primers for MAST1 and divergent primers for circMAST1 and found that the circular form of circMAST1 could only be amplified from cDNA instead of gDNA (Fig. 1D). Besides, results showed that circMAST1 was resistant to RNase R treatment, whereas the linear MAST1 was digested (Fig. 1E and Additional file 1: Fig. S1A). Since circular RNAs do not possess a 3´-poly adenylated tail, we used random primers, or oligo dT primers to synthesize reverse transcript products from SiHa and HeLa cells. Results showed that circMAST1 was only been detectable in cDNA from random primers (Fig. 1F). At certain time points, the expression of circMAST1 had higher stability than MAST1 mRNA following actinomycin D treatment (Fig. 1G and Additional file 1: Fig. S1B). To measure the subcellular localization of circMAST1, we performed qRT-PCR analysis to measure cytoplasmic and nuclear circMAST1 respectively. The results demonstrated that circMAST1 was localized both in the nucleus and cytoplasm of SiHa and HeLa cells (Fig. 1H, I), which were further confirmed by FISH assay (Fig. 1J). Collectively, these findings confirmed that circMAST1 is a circular RNA localized both in the nucleus and cytoplasm.

### Decreased expression of circMAST1 was associated with a poor prognosis in patients with CCa

To investigate the clinical significance of circMAST1 in patients with CCa, we quantified circMAST1 expression in 42 CCa and 17 normal cervical tissues. CircMAST1 was significantly downregulated in CCa tissues compared with normal cervical tissues (Fig. 1K). Meanwhile, we found that circMAST1 had a lower expression in CCa tissues with lymph node metastasis (LNM) than in CCa tissues without LNM (Fig. 1L). Next, we compared the expression of circMAST1 in six cervical cancer cell lines and a normal cervical cell line H8. Results showed that circMAST1 expression levels were higher in normal cervical cell line than in the cancer cell lines (Additional file 1: Fig. S1C). Moreover, the relationship between circMAST1 expression and the clinicopathological characteristics of patients with CCa was investigated (Additional file 1: Table S4). we found that lower expression levels of circMAST1 in primary CCa samples were associated with certain clinical factors, including tumor size (P = 0.0004) and lymph node metastasis (P = 0.0169). Remarkably, patients with low expression levels of circMAST1 showed poor overall survival (OS) as depicted by the Kaplan-Meier survival analysis (Fig. 1M). Together, our results supported that circMAST1 expression levels were reduced in CCa tissues, especially those with lymph node metastasis. Further analysis revealed that low expression of circMAST1 was associated with CCa progression and suggested poor prognosis.

### CircMAST1 inhibited the tumor growth and lymph node metastasis of CCa

**Fig. 2:**
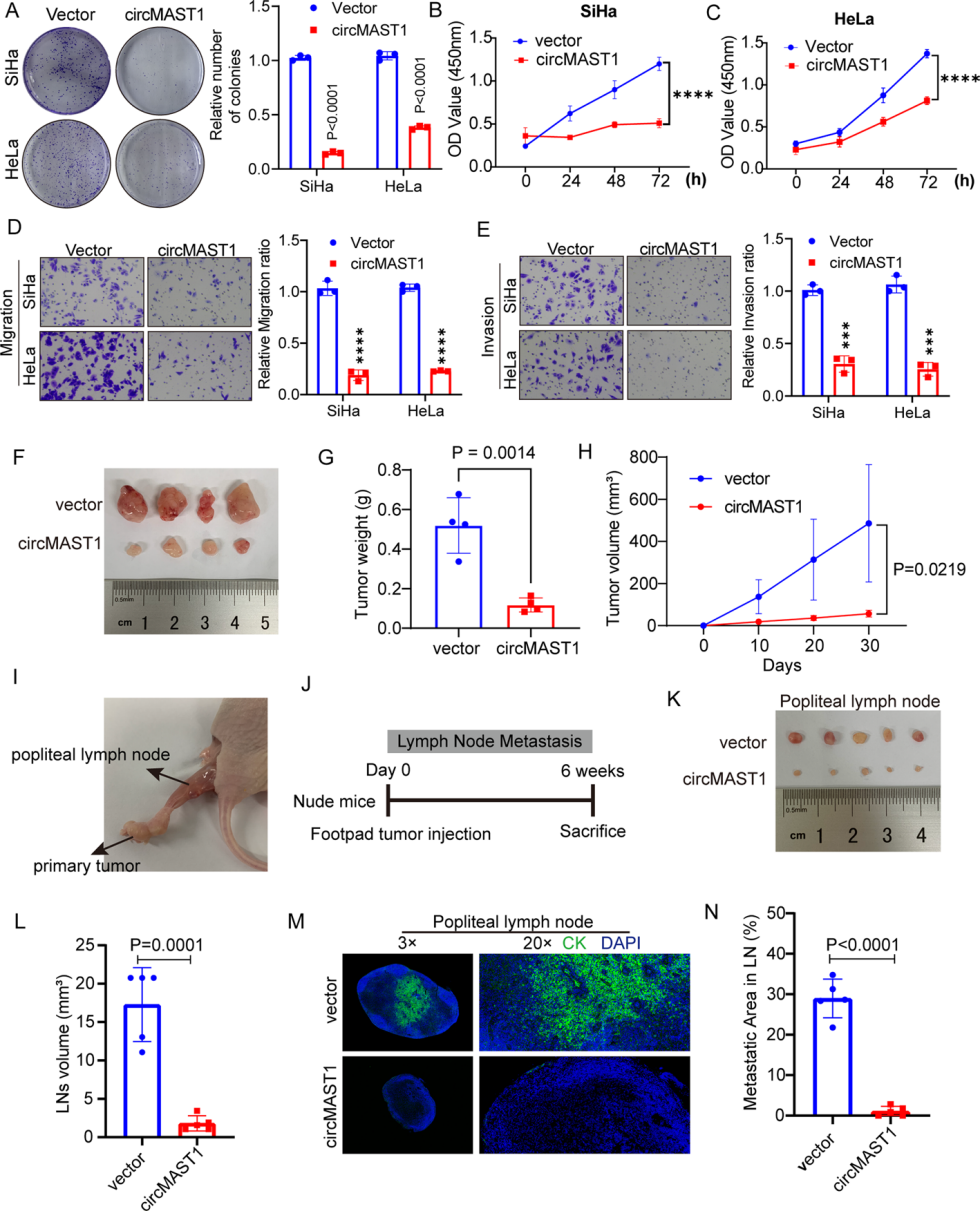
circMAST1 suppresses tumor growth and metastasis in CCa in vitro and in vivo.** A**-**C** The proliferative abilities of SiHa and HeLa cells were measured by the colony formation (A) and CCK-8 assay (B, C) after the overexpression of circMAST1. **D**,** E** Migration and invasion assays for SiHa and HeLa cells with circMAST1 overexpression. Original magnification, ×100. **F** Representative images of dissected tumors from nude mice transplanted with stable circMAST1 overexpression. **G** The mean weight of tumors upon sacrifice in the experimental groups. **H** Subcutaneous tumor growth curves of mice in different treatment groups. **I**,** J** The indicated CCa cells were injected into the footpads of the nude mice, and the popliteal LNs were enucleated and analyzed after 6 weeks of injection. **K**, **L** Representative images of enucleated popliteal LNs (K) and histogram analysis of the LN volume (L) in the indicated cells. **M** Representative images of immunostaining of CK as indicators of LN status. CK (pan-cytokeratin) was used as the marker of tumor cells. **N** Quantification of metastatic area of popliteal LN metastasis in the indicated groups. ***P < 0.001. ****P < 0.0001

We selected commonly used SiHa and HeLa cells to perform gain- and loss-of-function assays to investigate the effect of circMAST1 on the progression of CCa. The transfection of circMAST1-overexpressing plasmids, or shRNAs targeting the back-splice region, efficiently overexpressed or silenced circMAST1 in CCa cells (Additional file 1: Fig. S2A, B). Next, colony formation assay and CCK-8 assay demonstrated that circMAST1 overexpression significantly suppressed cell proliferation (Fig. 2A-C). Furthermore, Transwell cell assays demonstrated that the ectopic expression of circMAST1 dramatically reduced the migration and invasiveness of CCa cells (Fig. 2D, E). Conversely, circMAST1 depletion increased the in vitro proliferation, migration, and invasiveness of SiHa and HeLa cells (Additional file 1: Fig. S2C-I). We also employed a series of animal experiments to confirm the effects of circMAST1 on tumor growth and metastasis in vivo. A subcutaneous xenograft tumor model demonstrated that circMAST1 overexpression significantly decelerated tumor growth, evidenced by the smaller tumor volume and weight compared with control cells (Fig. 2F-H, Additional file 1: Fig. S3A). A nude mouse model of lymph node metastasis was employed to explore the effect of circMAST1 on lymphatic metastasis of cervical cancer (Fig. 2I, J). Popliteal lymph nodes were harvested and analyzed at the given time point after CCa cells were implanted into the footpads of nude mice. CircMAST1 overexpression remarkably inhibited lymph node metastasis. Compare with the control group, smaller volume of popliteal lymph nodes was obtained from circMAST1 tumor group (Fig. 2K, L). Moreover, we used mmunostaining of pan-cytokeratin to confirmed the metastatic CCa cells in lymph node, and confirmed that circMAST1 significantly prevented lymph node metastasis of cervical cancer (Fig. 2M, N). In summary, our results revealed that circMAST1 impaired the malignant progression of CCa both in vitro and in vivo.

### YAP was a downstream target for circMAST1

**Fig. 3:**
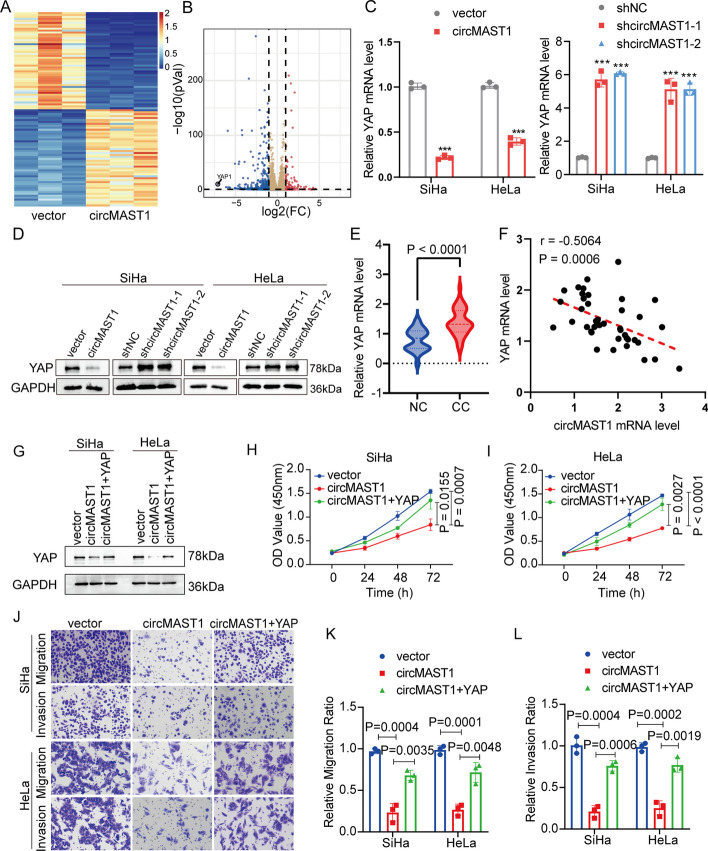
YAP is a downstream target of circMAST1.** A**,** B** RNA-seq analysis revealed the differentially expressed genes after the overexpression of circMAST1 in SiHa cells. **C**,** D** YAP mRNA and protein expression levels were analyzed by qRT-PCR and western blotting after circMAST1 overexpression (left) or ablation (right). **E** Expression levels of YAP in CCa tissues in comparison with normal tissues, as determined by qRT-PCR. **F** Correlation analysis of mRNA expression levels between circMAST1 and YAP in CCa tissues. **G** Protein expression levels of YAP were analyzed by western blotting in the indicated groups. **H**,** I** Cell proliferation ability was detected by CCK-8 in the indicated groups. **J**–**L** Transwell assays were used to detect the migration and invasion capabilities of the indicated cells. Original magnification, × 100

We next performed RNA-seq analysis to further investigate the molecular mechanisms by which circMAST1 suppresses the proliferation and aggressiveness of CCa cells. Of differentially expressed downstream genes of circMAST1, YAP was the most downregulated gene in SiHa cells after circMAST1 overexpression (Fig. 3A, B). Besides, we used qRT-PCR and immunoblotting, which validated that the expression levels of YAP were significantly downregulated by circMAST1 overexpression and dramatically upregulated after circMAST1 knockdown in CCa cells (Fig. 3C, D). In addition, we validated YAP was upregulated in CCa samples compared with normal cervical tissues using qRT-PCR (Fig. 3E). We also detected the correlation between circMAST1 and YAP in CCa specimens. We found that the expression level of circMAST1 was significantly and negatively correlated with the mRNA levels of YAP (*r* = −0.5064, *P* = 0.0006) (Fig. 3F). Therefore, YAP was considered a potential downstream target for circMAST1.

### CircMAST1 suppressed tumor growth and metastasis in CCa by regulating YAP expression

**Fig. 4:**
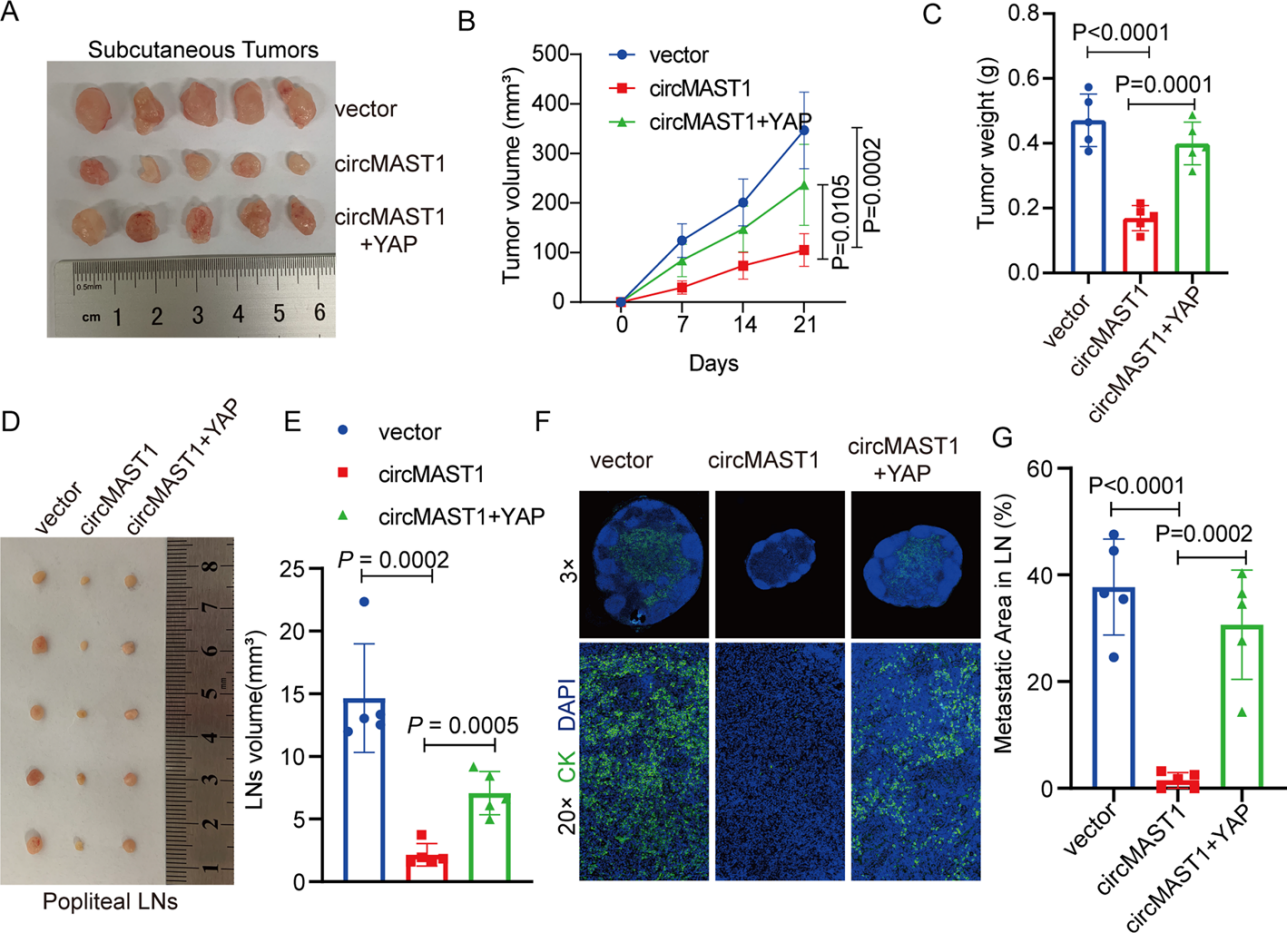
circMAST1 suppresses tumor growth and metastasis in CCa via regulating YAP in vivo.** A** The image of dissected tumors from nude mice transplanted with stably overexpressed circMAST1 CCa cells or circMAST1 overexpression/YAP overexpression cells. **B** Subcutaneous tumor growth curves of mice in different treatment groups. **C** The mean weight of tumors upon sacrifice in the experimental groups. **D**,** E** Representative images of enucleated popliteal LNs (**D**) and histogram analysis of the LN volume (**E**) in the indicated cells. **F** Representative images of IF as indicators of LN status. **G** Quantification of metastatic area of popliteal LN metastasis in the indicated groups

Then, we extensively investigate whether circMAST1 blocked the proliferation and invasion of CCa cells via a YAP-dependent manner. CCK-8 assay indicated that YAP overexpression abrogated the suppressive effects of circMAST1 on cell proliferation (Fig. 3G–I). Ectopic expression of YAP also rescued the migration and invasion abilities of SiHa and HeLa cells overexpressing circMAST1 (Fig. 3J–L). Moreover, we conducted animal experiments to further confirm the the effects of circMAST1 on tumor growth and metastasis. Results from the subcutaneous xenograft tumor model indicated that circMAST1 overexpression suppressed tumor growth, while YAP overexpression partially reversed the inhibitory effect of circMAST1 overexpression on tumor growth (Fig. 4A). Furthermore, YAP overexpression partially increased the number of Ki-67-positive cells compared with circMAST1 overexpression in subcutaneous tumors (Additional file 1: Fig. S3B). Moreover, YAP overexpression partially attenuated the effects of circMAST1 overexpression on tumor weight and size (Fig. 4B, C). We used a lymph node metastasis model to explore the effects of circMAST1 and YAP in lymph node metastasis of CCa. We found that the volume of the popliteal lymph nodes was significantly smaller in the circMAST1 overexpression group than in the control group. The YAP overexpression group showed a greater nodal volume than the circMAST1 overexpression group (Fig. 4D, E). Cytokeratin immunostaining confirmed that circMAST1 overexpression significantly inhibited the metastatic potential of CCa cells to popliteal lymph nodes, which was reversed by YAP overexpression (Fig. 4F, G). Collectively, these results indicated that circMAST1 suppressed the growth and lymph node metastasis of CCa by regulating the expression of YAP.

### CircMAST1 directly interacted with NAT10

**Fig. 5:**
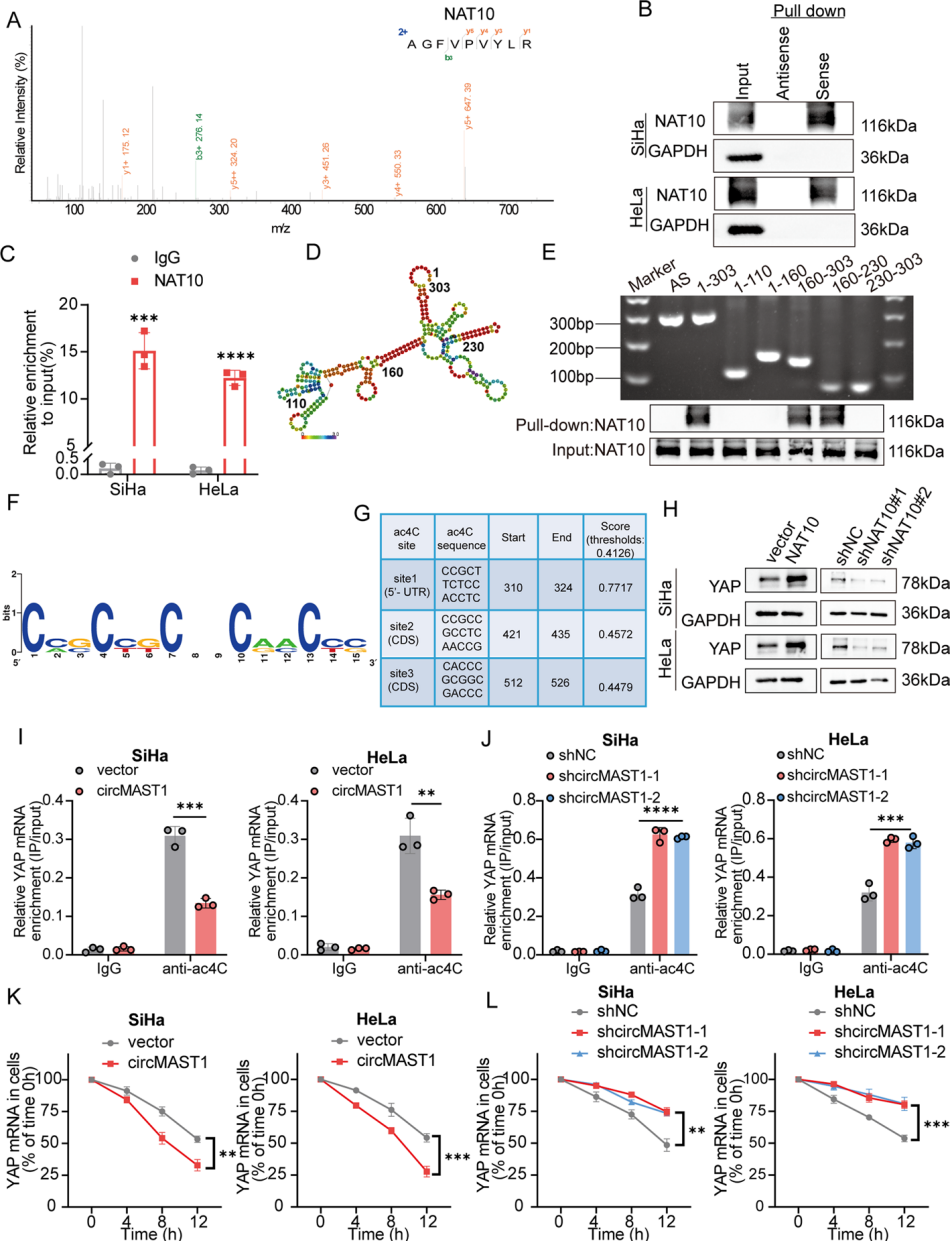
circMAST1 binds with NAT10 and regulates ac4C modification of YAP mRNA.** A** Mass spectrometry identified NAT10 was pulled down by circMAST1 from SiHa cells lysates. **B**,** C** The interaction between circMAST1 and NAT10 in SiHa and HeLa cells was shown by pull-down assay (**B**) and RIP assay (**C**). **D** The predicted secondary structure of circMAST1 using the RNAfold WebServer, based on the minimum free energy. Color scales indicated the confidence of predictions for each base, and the red shades demonstrated the predictions with strong confidence. **E** RNA pulldown assay showed NAT10 pulled down by biotin-labeled circMAST1 of different lengths. **F**,** G** Prediction of conserved acetylation site on YAP mRNA by PACES tool. **H** Relative protein expression levels of YAP were analyzed by western blotting after NAT10 overexpression or knockdown. **I**,** J** The regulatory role of circMAST1 on YAP ac4C in SiHa and HeLa cells confirmed by the acRIP-qPCR assay in the indicated groups. **K**,** L** The impact of circMAST1 on YAP mRNA stability confirmed by the RNA decay assay. Each experiment was performed at least three times independently. **P < 0.01. *** *P* < 0.001. ****P < 0.0001

We investigated the mechanisms by which circMAST1 repressed YAP expression. Previous studies showed that circRNAs can sponge protein and subsequently modulate the expression of target genes [26, 27]. We performed an RNA pull-down assay to identify circMAST1-binding proteins using mass spectroscopy. We found that among the top-ranked putative binding proteins of circMAST1, NAT10, the only enzyme responsible for mRNA ac4C modification [19], was identified and validated to interact with circMAST1(Fig. 5A). RNA immunoprecipitation (RIP) assay further confirmed the specific interaction of NAT10 with circMAST1 (Fig. 5B, C). Moreover, various truncated constructs of circMAST1 molecules and RNA pull-down assay revealed that NAT10 protein markedly interacted with segment 160–230 nt of circMAST1 (Fig. 5D, E). Besides, circMAST1 overexpressing and knockdown barely affected the mRNA and protein levels of NAT10 (Additional file 1: Fig. S4A-C), suggesting that circMAST1 may participate in the regulation of NAT10-mediated YAP mRNA ac4C modification, which has not been verified to date. In summary, these results indicated that circMAST1 could directly bind to NAT10.

### NAT10 improved the YAP mRNA ac4C modification and stability in CCa cells

YAP is a target for certain types of post-transcriptional modification and plays important roles in cancer progression [28, 29]; however, whether YAP mRNA can be modified by NAT10-mediated ac4C still remains unknown. Three highly conserved acetylation sites on YAP mRNA were predicted by the PACES tool (Fig. 5F, G). Our results showed that the YAP mRNA level was significantly upregulated by NAT10 overexpression in SiHa and HeLa cell lines. Consistently, NAT10 depletion reduced YAP mRNA level (Additional file 1: Fig. S5A-C). Similar results were also observed at the protein level (Fig. 5H). Based on these results, we predicted that NAT10 regulates YAP expression via ac4C modification at the mRNA level. Subsequently, ac4C RIP-qPCR showed that ac4C-acetylated YAP mRNA levels decreased in NAT10-knockdown CCa cells (Additional file 1: Fig. S5D) and increased in NAT10-overexpressing CCa cells (Additional file 1: Fig. S5E). In addition, we performed an mRNA stability assay to investigate the effects of NAT10-mediated ac4C modification on YAP mRNA. We observed that NAT10 overexpression decelerated the degradation of YAP mRNA in SiHa and HeLa cell lines (Additional file 1: Fig. S5F), whereas NAT10 knockdown accelerated the degradation of YAP mRNA after treatment with actinomycin D, a transcriptional inhibitor (Additional file 1 Fig. S5G). Consistently, previous studies reported that NAT10 can regulate the MDM2 mRNA stability in gastric cancer [30]. Furthermore, compared with normal cervix tissues, IHC staining demonstrated that YAP exhibited markedly higher protein levels in cervical cancer tissues (Additional file 1: Fig. S5H). Additionally, a positive correlation was observed between NAT10 protein expression and YAP protein expression in IHC staining (Additional file 1 Fig. S5I, J). Take together, our results confirmed that NAT10 regulated YAP mRNA stability and expression in an ac4C-dependent manner.

### CircMAST1 blocked NAT10-mediated ac4C modification of YAP mRNA

We used ac4C-RIP to detect the ac4C modification level of YAP mRNA after the gain and loss of function of circMAST1 to investigate whether circMAST1 repressed YAP expression in a NAT10-dependent manner. We found that circMAST1 overexpression decreased YAP mRNA ac4C modification, and circMAST1 ablation enhanced YAP mRNA ac4C modification (Fig. 5I, J). Moreover, a significant decrease in YAP mRNA stability was evident in the circMAST1 overexpression group compared with the control group (Fig. 5K). Conversely, the loss of function of circMAST1 markedly decelerated YAP mRNA degradation in both SiHa and HeLa cells (Fig. 5L). Together, these data suggest the existence of a causal relationship between circMAST1 and NAT10-mediated ac4C modification of YAP mRNA in CCa cells.

**Fig. 6:**
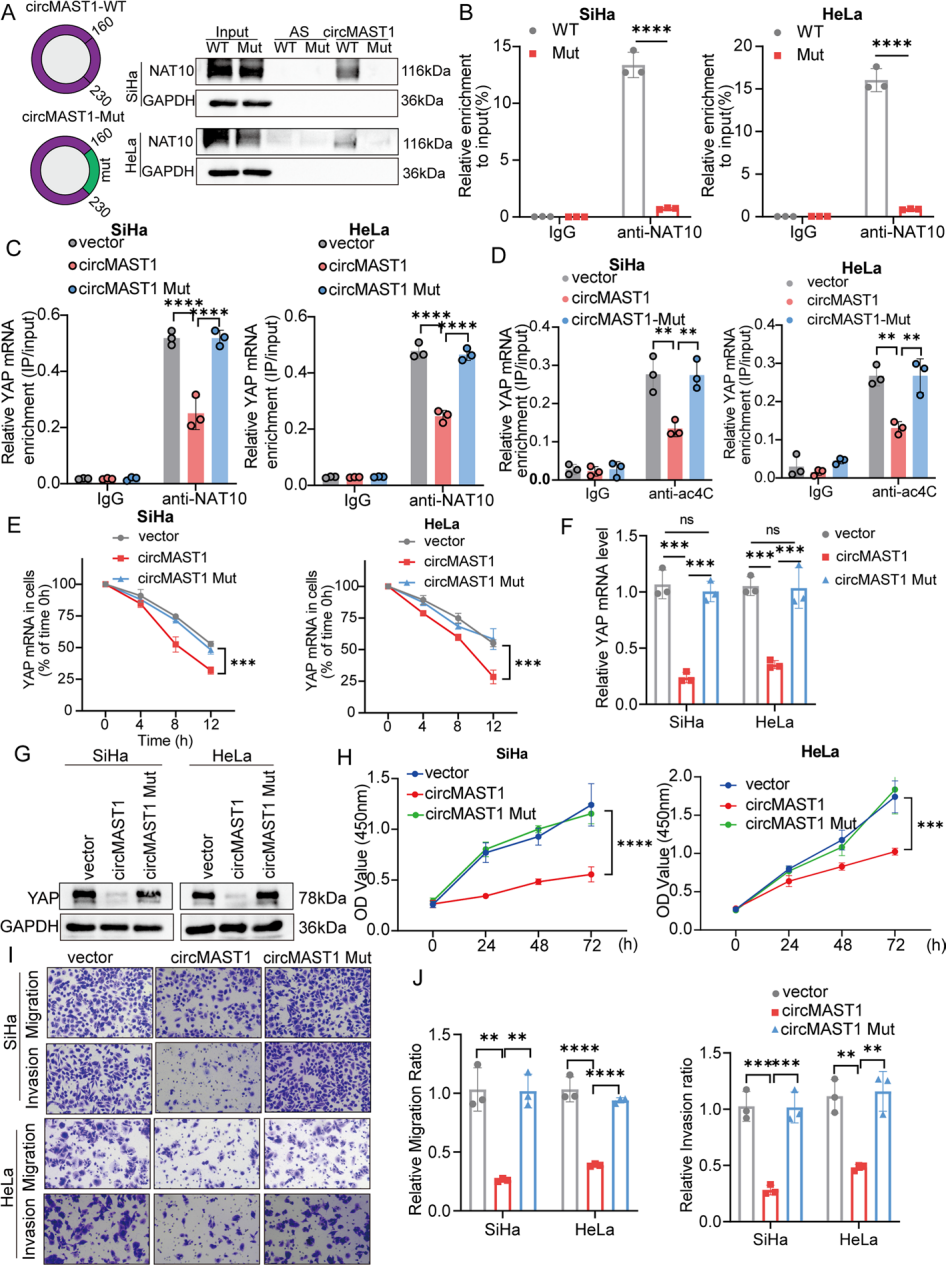
circMAST1 blocks NAT10 from binding to and ac4C modifying YAP mRNA.** A**,** B** Diagram for circMAST1 mutation plasmid construction (**A**, left). The interaction between circMAST1 and NAT10 in SiHa and HeLa cells shown by pull-down assay (A, right) and RIP assay (**B**) when transfection with the wild type or mutant circMAST1. **C** RIP assays were used to measure the recruitment of NAT10 protein to YAP mRNA in the indicated groups. **D** Ectopic expression of circMAST1 abrogated YAP mRNA ac4C modification determined by ac4C-RIP-qPCR experiment, whereas circMAST1 mutant could not show appreciate effect. **E** The YAP mRNA stability confirmed by the RNA decay assay in WT and Mut circMAST1 groups of SiHa and HeLa cells. **F**,** G** YAP mRNA and protein expression levels were analyzed by qRT-PCR and western blotting in the indicated groups. **H** The proliferative abilities of SiHa and HeLa cells were measured by the CCK-8 assay. **I**,** J** Migration and invasion assays for SiHa and HeLa cells. Original magnification, ×100. Each experiment was performed at least three times independently. **P < 0.01. ***P < 0.001. ****P < 0.0001

Since circMAST1 could bind to NAT10 and repress YAP ac4C modification and mRNA stability, circMAST1 might sequester NAT10 and block NAT10-meidataed YAP mRNA modification. Thus, we constructed a mutant circMAST1 (160-230nt) that could not bind to NAT10 (Fig. 6A). We conducted an anti-NAT10 RIP experiment and wild-type circMAST1 significantly decreased the interaction between NAT10 and YAP mRNA compared with mutant circMAST1 (Fig. 6B). In line with our previous results, ac4C-RIP-qPCR experiment illuminated that ectopic expression of circMAST1 abrogated YAP mRNA ac4C modification, whereas mutant circMAST1 could not show the same effect (Fig. 6C, D). Besides, YAP mRNA degradation speed increased after treatment with wild type circMAST1 but not mutant circMAST1 (Fig. 6E). Moreover, mutant circMAST1 did not inhibit YAP expression relative to the wild-type circMAST1 in both mRNA and protein levels (Fig. 6F, G). Furthermore, in vitro assays indicated that mutant circMAST1 could not inhibit the proliferation and invasion of CCa cells, whereas wild-type circMAST1 remarkably suppressed tumor cell growth and metastasis (Fig. 6H–J and Additional file 1: Fig. S6A, B). These results support the concept that circMAST1 competitively sequesters NAT10 to decrease YAP mRNA ac4C modification and promote YAP mRNA degradation, thereby inhibiting the growth and metastasis of CCa.

## Discussion

In this study, we found that circMAST1 inhibited cervical tumor growth and metastasis by regulating the NAT10/YAP axis. Cervical cancer is one of the most common cancers among females, and the outcome of treatment is not satisfactory, especially among patients with advanced disease, large tumor size, and lymph node metastasis. Recently, numerous circRNAs have been identified by circRNA microarray analysis and high-throughput sequencing. However, the biological functions of circRNAs in CCa are still unknown, and the precise mechanisms circRNAs performed on development of CCa required further elucidated. In this study, we identified a circRNA named circMAST1, which played a significant repressive effect on the progression of CCa. Then, we found that circMAST1 was downregulated in cervical cancer cell lines and tissues compared with normal cervical cells and tissues. Furthermore, our results identified that circMAST1 downregulation markedly correlated with tumor size, LVSI, LN metastasis, and poor survival, highlighting its value as a novel prognostic biomarker for patients with cervical cancer.

Large primary tumor size and the occurrence of lymph node metastasis confer a poor prognosis in patients with CCa. Therefore, a deep understanding of the molecular mechanisms underlying the growth and metastasis of CCa may help identify high-risk patients and provide an effective therapeutic target. In this study, in vitro functional assays supported that circMAST1 inhibited CCa cell proliferation, migration, and invasion. Moreover, the in vivo model verified that circMAST1 suppressed the subcutaneous tumor formation ability of CCa cells, while loss of circMAST1 exhibited the opposite effect. Footpad transplantation experiments in nude mice were performed to assess lymph node metastasis ability. The results indicated that circMAST1 overexpression inhibited CCa cell metastasis to lymph nodes, evidenced by smaller LN volume and smaller metastatic areas in LNs. Therefore, low circMAST1 expression is a favorable condition for LN metastasis in cervical cancer.

YAP is the critical downstream regulatory target in the Hippo signaling pathway, which has been reported to participate in various diseases, including cervical cancer [31–36]. It was reported that YAP expression is associated with a poor prognosis of cervical cancer, and high YAP expression induces CCa cell proliferation and migration [34]. Here, we discovered that YAP is a downstream target of circMAST1. We performed RNA-Seq after circMAST1 overexpression in SiHa cells to obtain a deeper insight into how circMAST1 inhibits CCa progression. The results suggested that YAP is a potential downstream target of circMAST1. Then, we verified that circMAST1 can suppress YAP mRNA and protein expression in CCa cells. In vitro and in vivo functional experiments also confirmed that YAP overexpression attenuated the inhibitory effects of circMAST1 on the proliferation and metastasis of CCa cells, suggesting YAP as a target of circMAST1. Furthermore, a significantly negative correlation was found between circMAST1 and YAP mRNA levels in CCa tissues. Therefore, our study highlighted the suppressive role of the circMAST1/YAP axis in CCa tumor growth and lymph node metastasis.

We verified that a circRNA can influence NAT10-mediated mRNA ac4C modification. Post-transcriptional modification on the RNA level is critical to RNA structure and function [37, 38]. However, previous studies mainly focused on m^6^A, and other types of RNA modification, such as ac4C, have rarely been noticed [39]. Ac4C modification is a type of conserved RNA modification mainly occurring in tRNA and 18S rRNA [20]. Since Arango *et al.* first reported the effects of ac4C modification on mRNA stability and translation efficiency, studies on mRNA levels have received more attention [19, 40–42]. As an ac4C “writer”, NAT10 is the only reported enzyme for ac4C modification, which is also an important regulator for tumorigenesis and metastasis [23, 43, 44]. Numerous studies revealed that post-transcriptional modification regulates the activity and subcellular localization of YAP. For instance, circ-ZNF609 regulated heart repair via controlling YTHDF1- and YTHDF2-mediated YAP m6A modification [39]. In addition, NSUN2 and ALYREF promoted YAP m5C modification in its 3’-UTR regions, increasing mRNA stability in lung adenocarcinoma [45]. In this study, we explored the effect of circMAST1 on ac4C modification of YAP mRNA, and the results uncovered that circMAST1 hindered NAT10 binding to YAP mRNA, thereby decreasing YAP mRNA ac4C modification and degradation. Consistent with our findings, recent studies have reported that a novel lncRNA LINC00623 binds to NAT10 and prevents its ubiquitination-dependent degradation, thus influencing mRNA ac4C modification and expression in pancreatic cancer [23]. Here, we identified a new mechanism by which circMAST1 decreased YAP ac4C modification in cervical cancer, suggesting a promising value of circMAST1 for treating patients with cervical cancer. In particular, these findings revealed that circRNA-mediated post-transcriptional modification of mRNA is a novel finding in clarifying the mechanism of CCa progression.

## Conclusions

**Fig. 7:**
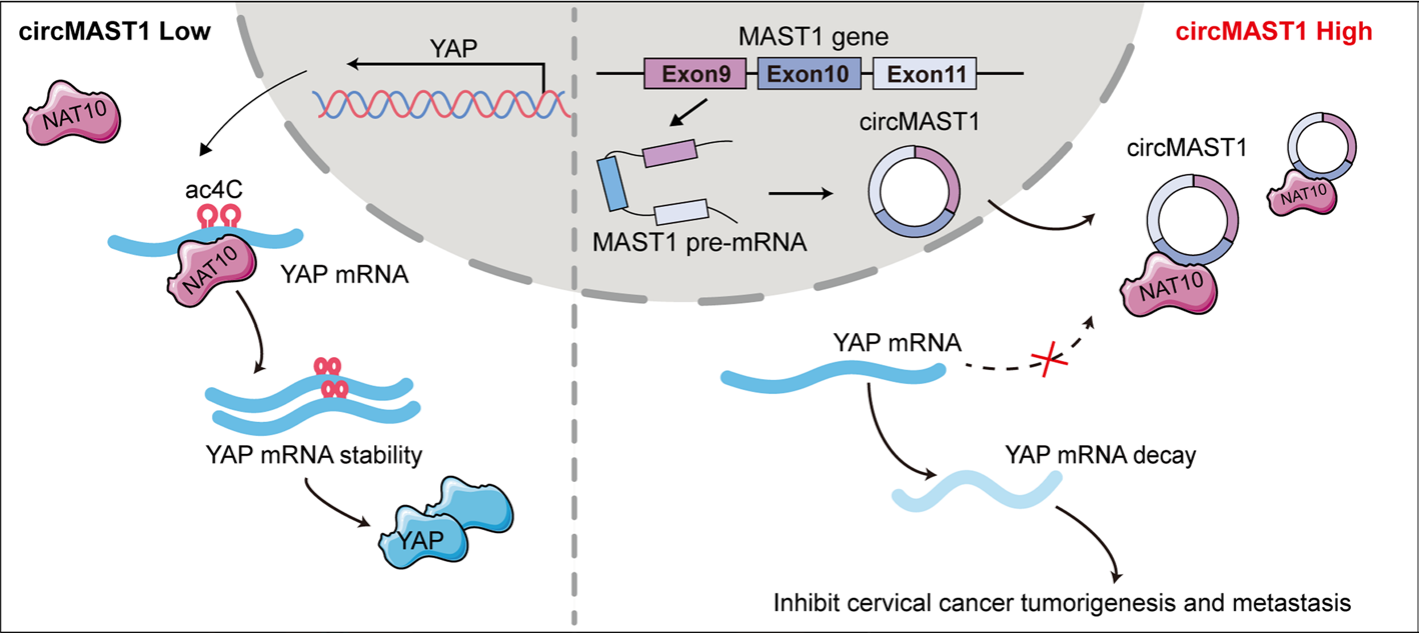
A schematic diagram of the mechanism. circMAST1 competitively sequesters NAT10 and decreases YAP mRNA ac4C modification to promote its mRNA degradation, resulting in inhibition of tumor growth and metastasis in CCa

In conclusion, our results revealed that circMAST1 inhibited NAT10 from binding to YAP mRNA and decreased YAP mRNA ac4C modification to promote its degradation and inhibit tumor growth and metastasis in CCa (Fig. 7). These findings expand our understanding of the mechanisms underlying CCa development. In particular, we identified the potential role of circMAST1 as a novel target for the future treatment of cervical cancer.

### Supplementary Information

**Additional file 1:** **Figure S1.**Characterization of circMAST1 in CCa cells. **A **The qRT-PCR analysis for the expression of circMAST1 and MAST1 mRNA after treatment with RNase R in HeLa cells. **B** qRT-PCR analysis for the expression of circMAST1 and MAST1 mRNA after treatment with actinomycin D at the indicated time points in HeLa cells. **C** Relative expression of circMAST1 in CCa cell lines and a normal cervix cell line H8. Each experiment was performed at least three times independently. ***P< 0.001. ****P < 0.0001. **Figure S2. **Knockdown circMAST1 promoted proliferation, migration, and invasion of CCa cells. **A** The expression levels of circMAST1 in SiHa and HeLa cells stably transfected with circMAST1 or corresponding negative control were detected by RT-qPCR. **B** The knockdown efficiency of circMAST1 using two different shRNAs in SiHa and HeLa cells. **C-F**  The proliferative abilities of SiHa and HeLa cells were measured by the CCK-8 assay (C, D) and colony formation assay (E, F) after the knockdown of circMAST1. **G-I** Migration and invasion assays for SiHa and HeLa cells with circMAST1 inhibition. Original magnification, ×100. Each experiment was performed at least three times independently. *P < 0.05. **P < 0.01, ***P < 0.001, ****P < 0.0001. **Figure S3. **Representative images of IHC staining for Ki-67 (**A**, **B**) and YAP (**B**) expression in tumor sections in the indicted groups. **Figure S4. **circMAST1 had no effect on NAT10 mRNA and protein levels. **A**–**C **NAT10 mRNA and protein expression levels were analyzed by qRT-PCR and western blotting after circMAST1 overexpression or ablation. Each experiment was performed at least three times independently. *ns* no significant. **Figure S5.** NAT10 improved the YAP mRNA ac4C modification and stability in CCa cells.** A** NAT10 protein expression levels were analyzed by western blotting after NAT10 overexpression or knockdown. **B**, **C** YAP mRNA levels were analyzed by qRT-PCR after NAT10 overexpression or ablation. **D**, **E** The acRIP followed by qPCR in the indicated groups of SiHa and HeLa cells. **F**, **G** The influence of NAT10 on YAP mRNA stability confirmed by the RNA decay assay. **H** NAT10 and YAP expression of IHC staining in normal cervix tissues and CCa tissues. **I**. The percentages of specimens with high or low levels of YAP in CCa with low or high expression of NAT10. **J** The correlation between YAP and NAT10 expression in CCa tissues was analyzed based on IHC score. Each experiment was performed at least three times independently. ***P < 0.001. ****P < 0.0001. **Figure S6.** Wild type circMAST1 but not Mutant circMAST1 suppressed CCa cells proliferation.** A**, **B** The proliferative abilities of SiHa and HeLa cells were measured by the colony formation in the indicated groups. Each experiment was performed at least three times independently. ***P < 0.001.****P < 0.0001; *ns* no significant. **Table S1.** Primers and oligos sequences. **Table S2.** Antibodies used in this study. **Table S3.** Mass spectroscopy result. **Table S4.** Correlation between circMAST1 expression and clinicopathologic characteristics of CCa. 

** Additional file 2:** Differentially Expressed CircRNAs in CCa.

## Data Availability

The datasets generated and/or analyzed during the current study are available from the corresponding author on reasonable request.

## Abbreviations

circRNA: circular RNA
circMAST1: hsa_circ_0049613
CCa: Cervical cancer
ac4C: N4-acetylcytidine
NAT10: N-acetyltransferase 10
YAP: Yes-associated protein
FISH: Fluorescence in situ hybridization
IHC: Immunohistochemistry
LNM: Lymph node metastasis
LN: Lymph node
FIGO: The International Federation of Gynecology and Obstetrics
LVSI: Lymphovascular space invasion
RIP: RNA immunoprecipitation
cDNA: Complementary DNA
gDNA: Genomic DNA
shRNAs: Short hairpin RNAs
actD: Actinomycin D
