# Supplementary material for: CircMAST1 inhibits cervical cancer progression by hindering the N4-acetylcytidine modification of YAP mRNA
Source: Cell Mol Biol Lett. 2024 Feb 8;29:25. doi: 10.1186/s11658-024-00540-6 (PMC10854152; doi:10.1186/s11658-024-00540-6)
Supplement: Supplementary file 1 — Additional file 1: Figure S1.Characterization of circMAST1 in CCa cells. A The qRT-PCR analysis for the expression of circMAST1 and MAST1 mRNA after treatment with RNase R in HeLa cells. B qRT-PCR analysis for the expression of circMAST1 and MAST1 mRNA after treatment with actinomycin D at the indicated time points in HeLa cells. C Relative expression of circMAST1 in CCa cell lines and a normal cervix cell line H8. Each experiment was performed at least three times independently. ***P< 0.001. ****P < 0.0001. Figure S2. Knockdown circMAST1 promoted proliferation, migration, and invasion of CCa cells. A The expression levels of circMAST1 in SiHa and HeLa cells stably transfected with circMAST1 or corresponding negative control were detected by RT-qPCR. B The knockdown efficiency of circMAST1 using two different shRNAs in SiHa and HeLa cells. C-F The proliferative abilities of SiHa and HeLa cells were measured by the CCK-8 assay (C, D) and colony formation assay (E, F) after the knockdown of circMAST1. G-I Migration and invasion assays for SiHa and HeLa cells with circMAST1 inhibition. Original magnification, ×100. Each experiment was performed at least three times independently. *P < 0.05. **P < 0.01, ***P < 0.001, ****P < 0.0001. Figure S3. Representative images of IHC staining for Ki-67 (A, B) and YAP (B) expression in tumor sections in the indicted groups. Figure S4. circMAST1 had no effect on NAT10 mRNA and protein levels. A–C NAT10 mRNA and protein expression levels were analyzed by qRT-PCR and western blotting after circMAST1 overexpression or ablation. Each experiment was performed at least three times independently. ns no significant. Figure S5. NAT10 improved the YAP mRNA ac4C modification and stability in CCa cells. A NAT10 protein expression levels were analyzed by western blotting after NAT10 overexpression or knockdown. B, C YAP mRNA levels were analyzed by qRT-PCR after NAT10 overexpression or ablation. D, E The acRIP followed by qPCR in the indicated gr [file 11658_2024_540_MOESM1_ESM.docx]

**
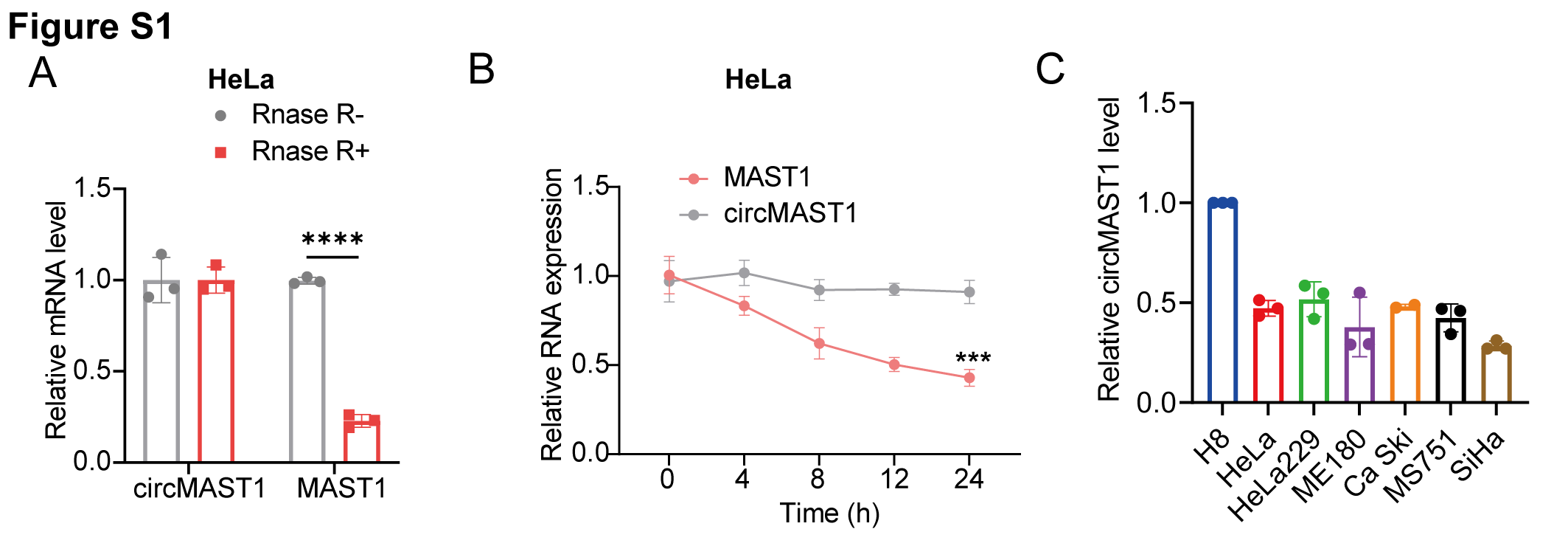
**

**Figure S1. Characterization of circMAST1 in CCa cells. A** The qRT-PCR analysis for the expression of circMAST1 and MAST1 mRNA after treatment with RNase R in HeLa cells. **B** qRT-PCR analysis for the expression of circMAST1 and MAST1 mRNA after treatment with actinomycin D at the indicated time points in HeLa cells. **C** Relative expression of circMAST1 in CCa cell lines and a normal cervix cell line H8. Each experiment was performed at least three times independently. ***P < 0.001. ****P < 0.0001.


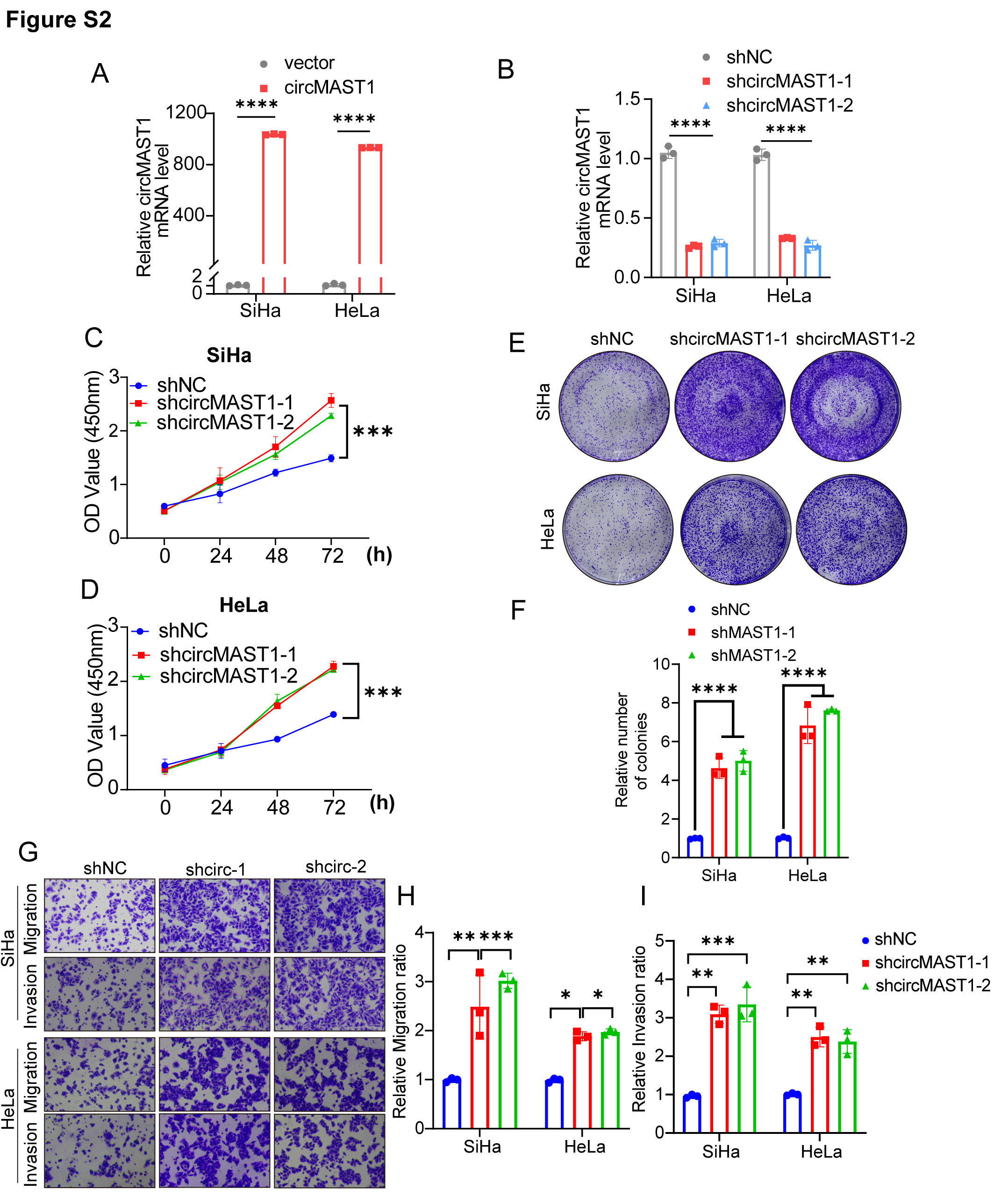


**Figure S2. Knockdown circMAST1 promoted proliferation, migration, and invasion of CCa cells. A** The expression levels of circMAST1 in SiHa and HeLa cells stably transfected with circMAST1 or corresponding negative control were detected by RT-qPCR. **B** The knockdown efficiency of circMAST1 using two different shRNAs in SiHa and HeLa cells. **C-F** The proliferative abilities of SiHa and HeLa cells were measured by the CCK-8 assay (C, D) and colony formation assay (E, F) after the knockdown of circMAST1. **G-I** Migration and invasion assays for SiHa and HeLa cells with circMAST1 inhibition. Original magnification, ×100. Each experiment was performed at least three times independently. *P < 0.05. **P < 0.01, ***P < 0.001, ****P < 0.0001.


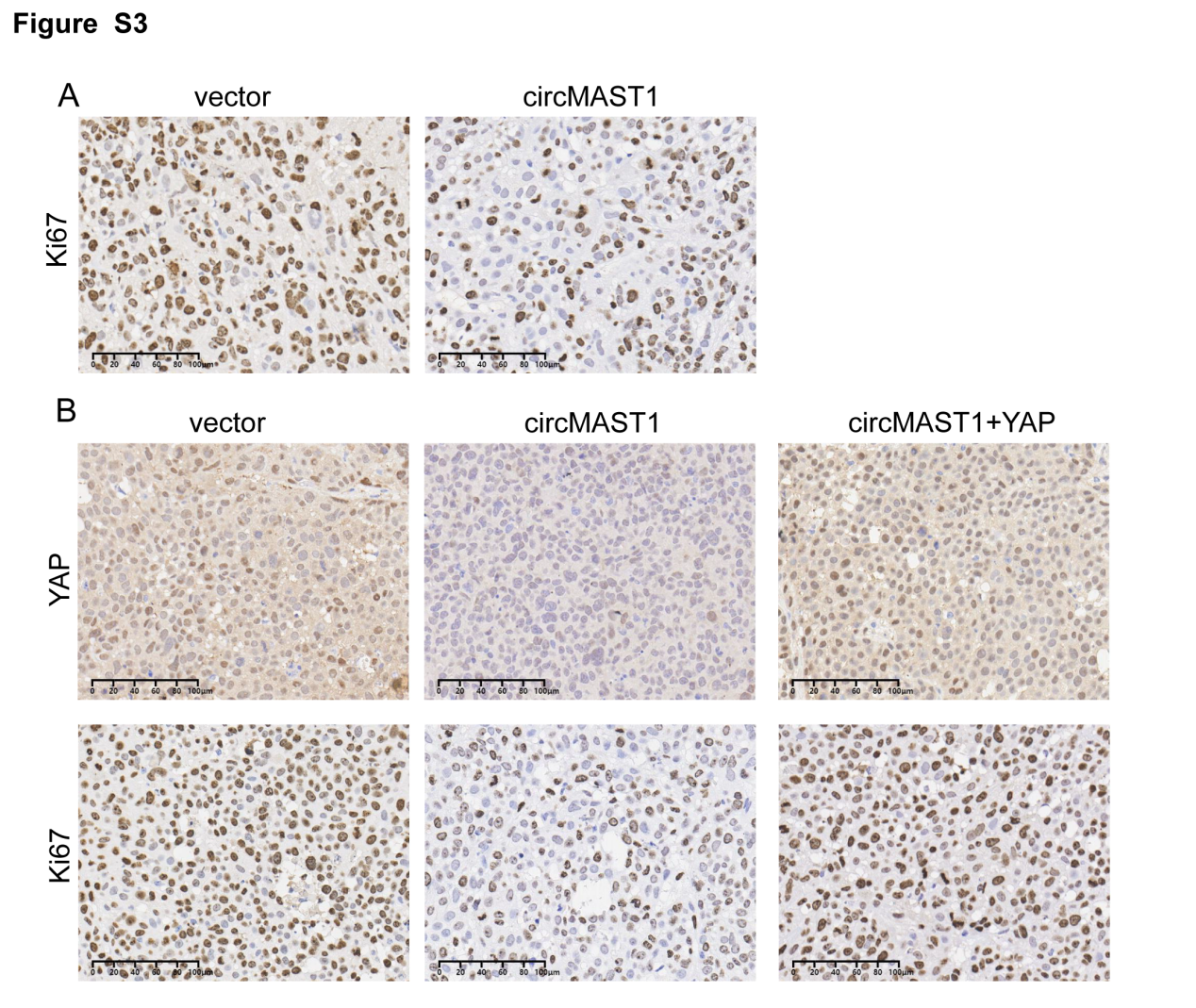


**Figure S3. Representative images of IHC staining for Ki-67 (A, B) and YAP (B) expression in tumor sections in the indicted groups.**

**
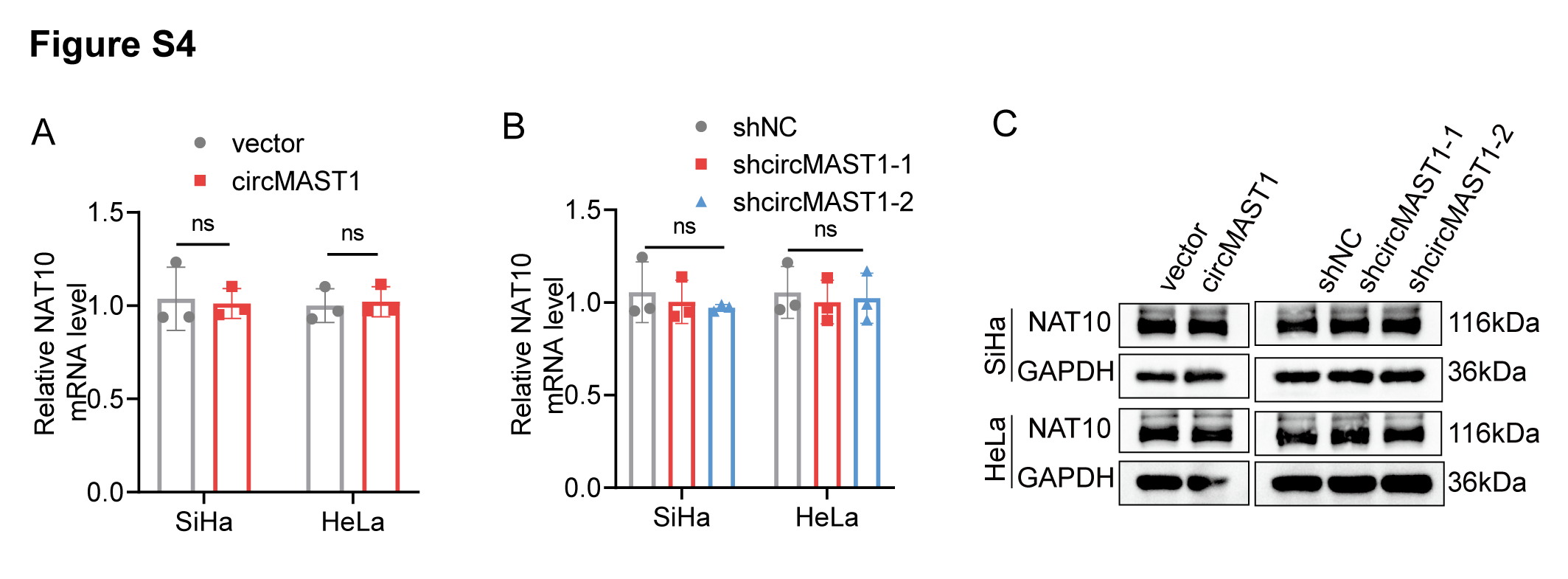
**

**Figure S4. circMAST1 had no effect on NAT10 mRNA and protein levels. A-C** NAT10 mRNA and protein expression levels were analyzed by qRT-PCR and western blotting after circMAST1 overexpression or ablation. Each experiment was performed at least three times independently. ns, no significant.


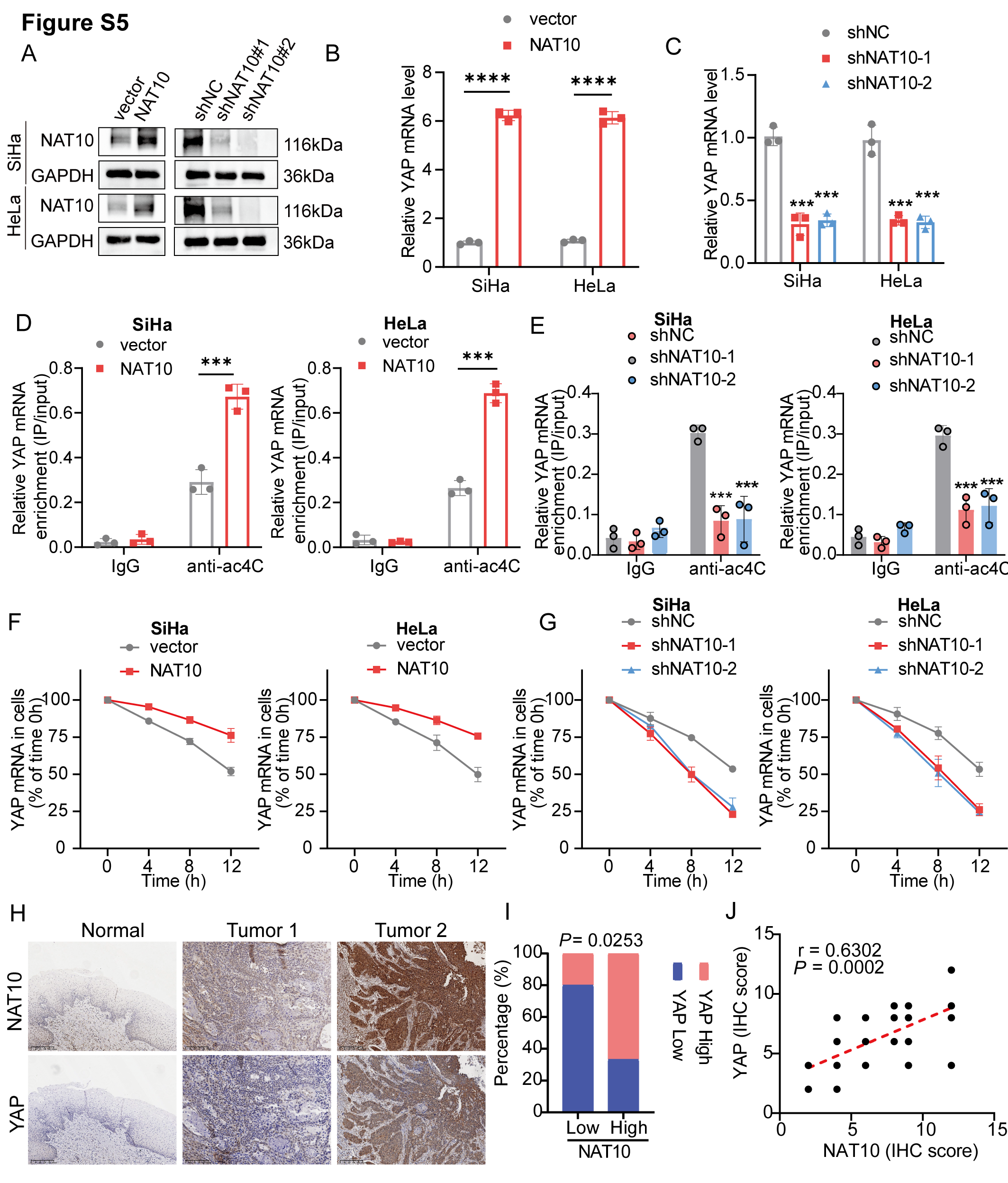


**Figure S5. NAT10 improved the YAP mRNA ac4C modification and stability in CCa cells. A** NAT10 protein expression levels were analyzed by western blotting after NAT10 overexpression or knockdown. **B, C** YAP mRNA levels were analyzed by qRT-PCR after NAT10 overexpression or ablation. **D, E** The acRIP followed by qPCR in the indicated groups of SiHa and HeLa cells. **F, G** The influence of NAT10 on YAP mRNA stability confirmed by the RNA decay assay. **H** NAT10 and YAP expression of IHC staining in normal cervix tissues and CCa tissues. **I**. The percentages of specimens with high or low levels of YAP in CCa with low or high expression of NAT10. **J** The correlation between YAP and NAT10 expression in CCa tissues was analyzed based on IHC score. Each experiment was performed at least three times independently. ***P < 0.001. ****P < 0.0001.


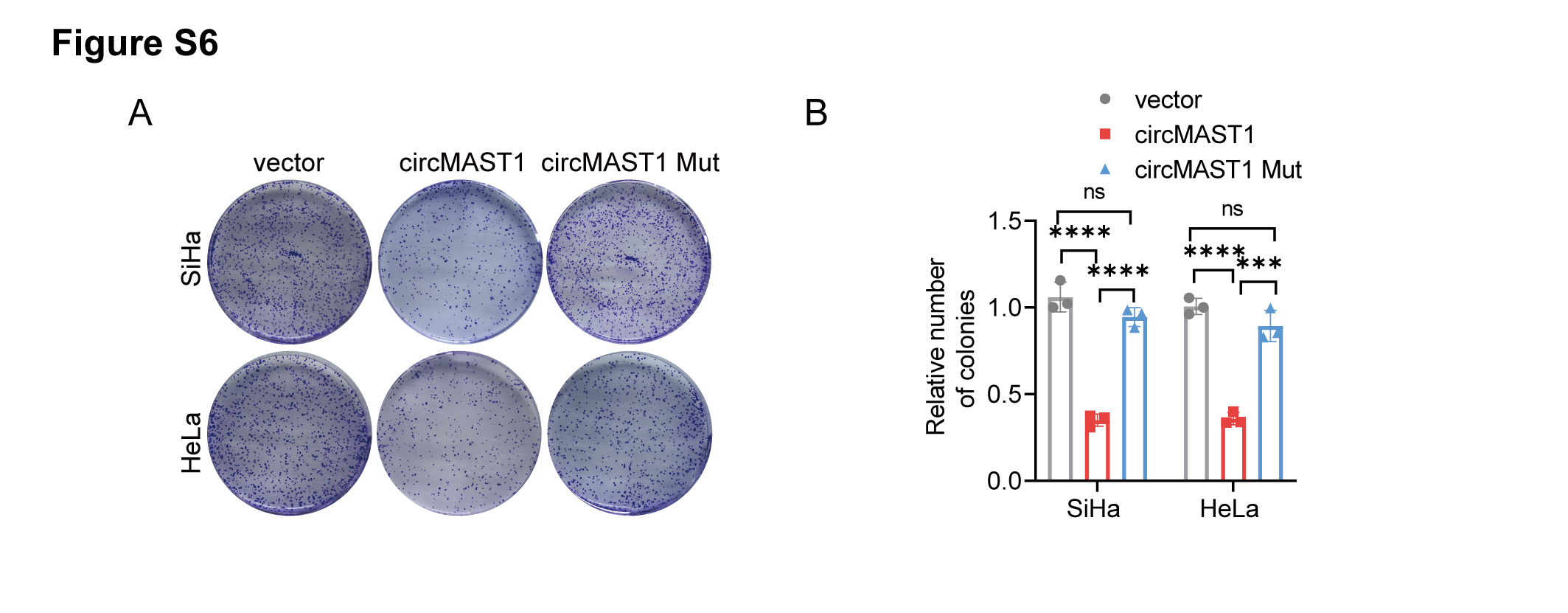


**Figure S6. Wild type circMAST1 but not Mutant circMAST1 suppressed CCa cells proliferation. A, B** The proliferative abilities of SiHa and HeLa cells were measured by the colony formation in the indicated groups. Each experiment was performed at least three times independently. ***P < 0.001. ****P < 0.0001; ns, no significant.

**Table S1. Primers and oligos sequences**

| NAT10 | **F** 5'-GCCTCTTGTAAGAAGTGTCTCG-3' |
| --- | --- |
|  | **R** 5'-TCTTTTCAGAGATGCCCTCGAT-3' |
| YAP | **F** 5'-TAGCCCTGCGTAGCCAGTTA-3' |
|  | **R** 5'-TCATGCTTAGTCCACTGTCTGT-3' |
| GAPDH | **F** 5'-AGAAGGCTGGGGCTCATTTG-3' |
|  | **R** 5'-AGGGGCCATCCACAGTCTTC-3' |
| CircMAST1 (divergent) | **F** 5'-ATCTGGAGGAACAGGACA-3' |
|  | **R** 5'-GCGTGAGATGATAATAAGCA-3' |
| CircMAST1 (convergent) | **F**  5'-CCTATGAACGCTCTGAGA-3' |
|  | **R** 5'-GTTGAATTCCAGGCACTC-3' |
| MAST1 | **F**  5'-TCTCTGGACCGCGCTTTCTA-3' |
|  | **R** 5'-TGAGGCTTTTCCGATTACTGGT-3' |
| U6 | **F** 5'-ACAGATCTGTCGGTGTGGCAC-3' |
|  | **R**  5'-GGCCCCGGATTATCCGACATTC-3' |
| circ0043280 | **F** 5'-CTACCTTTGACTCCTTGCTT-3' |
|  | **R**  5'-GACAGGAAAATCTGAAGTCATGT-3' |
| shcircMAST1-1 | **sense** 5’-CGATCTCTCTGAGGCCTATGA-3’ |
| shcircMAST1-2 | **sense** 5’-CTCTCTGAGGCCTATGAACGC-3’ |
| shNAT10-1 | **sense** 5’-GCAATTGTACACAGTGACTAT-3’ |
| shNAT10-2 | **sense** 5’-CGGCCATCTCTCGCATCTATT-3’ |
| FITC-labeled CircMAST1 probe | 5’-GCGTTCATAGGCCTCAGAGAGATC-3’ |
| **Primers for in *vitro* transcription** | |
| CircMAST1(1-303/mut) | **F** 5'-TAATACGACTCACTATAGGGGCCTATGAACGCTCTGAGAGCT-3' |
|  | **R** 5'-CTCAGAGAGATCGTCTTGCTCAGG-3' |
| CircMAST1(1-110) | **F** 5'-TAATACGACTCACTATAGGGGCCTATGAACGCTCTGAGAGCT-3' |
|  | **R** 5'-TTGAATTCCAGGCACTCCAGC-3' |
| CircMAST1(1-160) | **F** 5'-TAATACGACTCACTATAGGGGCCTATGAACGCTCTGAGAGCT-3' |
|  | **R** 5'-CCTTGGCGTGTCCTTCGG-3' |
| CircMAST1(160-303) | **F** 5'-TAATACGACTCACTATAGGGGAGGGCCACCTTGTGAAGACG-3' |
|  | **R** 5'-CTCAGAGAGATCGTCTTGCTCAGG-3' |
| CircMAST1(160-230) | **F** 5'-TAATACGACTCACTATAGGGGAGGGCCACCTTGTGAAGACG-3' |
|  | **R** 5'-AAGGGGTCACGGGTGAGGC-3' |
| CircMAST1(230-303) | **F** 5'-TAATACGACTCACTATAGGGTTCCAGATGTGGTGCATCTGG-3' |
|  | **R** 5'-CTCAGAGAGATCGTCTTGCTCAGG-3' |
| CircMAST1(antisense) | **F** 5'-TAATACGACTCACTATAGGGCGGATACTTGCGAGACTCTCG-3' |
|  | **R** 5'-GAGTCTCTCTAGCAGAACGAGTCCC-3' |

**Table S2. Antibodies used in this study**

| NAT10 | Proteintech, #13365-1-AP | WB (1:2000) |
| --- | --- | --- |
| YAP | Proteintech, #13584-1-AP | WB (1:5000), IHC (1:200) |
| GAPDH | Proteintech, #10494-1-AP | WB (1:10000) |
| HRP-conjugated Affinipure Goat Anti-Rabbit IgG(H+L) | Proteintech, #SA00001-2 | WB (1:10000) |
| HRP-conjugated Affinipure Goat Anti-Mouse IgG(H+L) | Proteintech, #SA00001-1 | WB (1:10000) |
| Pan-keratin | CST, #4545S | IHC (1:400) |
| Ki67 | Proteintech, #27309-1-AP | IHC (1:2000) |
| NAT10 | Abcam, #a**b194297** | IHC (1:500) |
| ac4C | Abcam, #a**b252215** | RIP (1:200) |

**Table S3. Mass spectroscopy result**

| RANK | prot_acc | prot_score | prot_mass | emPAI |
| --- | --- | --- | --- | --- |
| 1 | sp\|P62805\|H4_HUMAN | 128 | 11360 | 3.92 |
| 2 | sp\|P60709\|ACTB_HUMAN | 354 | 42052 | 3.21 |
| 3 | sp\|P23284\|PPIB_HUMAN | 199 | 23785 | 2.27 |
| 4 | sp\|P08729\|K2C7_HUMAN | 284 | 51411 | 2.26 |
| 5 | sp\|A0A075B6R9\|KVD24_HUMAN | 94 | 13128 | 2.19 |
| 6 | sp\|Q9H0A0\|NAT10_HUMAN | 22 | 116569 | 2.15 |
| 7 | sp\|P13645\|K1C10_HUMAN | 491 | 59020 | 2.12 |
| 8 | sp\|Q04695\|K1C17_HUMAN | 465 | 48361 | 2.07 |
| 9 | sp\|A0A075B6S2\|KVD29_HUMAN | 286 | 13249 | 1.51 |
| 10 | sp\|P60660\|MYL6_HUMAN | 119 | 17090 | 1.47 |
| 11 | sp\|P83731\|RL24_HUMAN | 168 | 17882 | 1.38 |
| 12 | sp\|P35908\|K22E_HUMAN | 342 | 65678 | 1.29 |
| 13 | sp\|P35268\|RL22_HUMAN | 90 | 14835 | 1.28 |
| 14 | sp\|P23396\|RS3_HUMAN | 172 | 26842 | 1.27 |
| 15 | sp\|P04264\|K2C1_HUMAN | 411 | 66170 | 1.17 |
| 16 | sp\|P62263\|RS14_HUMAN | 103 | 16434 | 1.12 |
| 17 | sp\|Q969Q0\|RL36L_HUMAN | 59 | 12746 | 1.04 |
| 18 | sp\|Q15208\|STK38_HUMAN | 189 | 54498 | 1.02 |
| 19 | sp\|P62851\|RS25_HUMAN | 55 | 13791 | 0.94 |
| 20 | sp\|Q5QNW6\|H2B2F_HUMAN | 60 | 13912 | 0.93 |
| 21 | sp\|P05787\|K2C8_HUMAN | 191 | 53671 | 0.92 |
| 22 | sp\|P08727\|K1C19_HUMAN | 161 | 44079 | 0.92 |
| 23 | sp\|P18124\|RL7_HUMAN | 100 | 29264 | 0.91 |
| 24 | sp\|P42766\|RL35_HUMAN | 94 | 14543 | 0.88 |
| 25 | sp\|P02533\|K1C14_HUMAN | 234 | 51872 | 0.85 |
| 26 | sp\|P62244\|RS15A_HUMAN | 65 | 14944 | 0.85 |
| 27 | sp\|Q71UM5\|RS27L_HUMAN | 30 | 9813 | 0.84 |
| 28 | sp\|P04259\|K2C6B_HUMAN | 136 | 60315 | 0.79 |
| 29 | sp\|Q07020\|RL18_HUMAN | 113 | 21735 | 0.77 |
| 30 | sp\|Q06830\|PRDX1_HUMAN | 60 | 22324 | 0.75 |
| 31 | sp\|P62249\|RS16_HUMAN | 56 | 16549 | 0.75 |
| 32 | sp\|P11142\|HSP7C_HUMAN | 240 | 71082 | 0.72 |
| 33 | sp\|O75688\|PPM1B_HUMAN | 113 | 53180 | 0.72 |
| 34 | sp\|Q9UNX3\|RL26L_HUMAN | 68 | 17246 | 0.71 |
| 35 | sp\|P62750\|RL23A_HUMAN | 86 | 17684 | 0.69 |
| 36 | sp\|Q9BQA1\|MEP50_HUMAN | 138 | 37442 | 0.66 |
| 37 | sp\|P35579\|MYH9_HUMAN | 731 | 227646 | 0.65 |
| 38 | sp\|P08779\|K1C16_HUMAN | 196 | 51578 | 0.64 |
| 39 | sp\|P05141\|ADT2_HUMAN | 62 | 33059 | 0.61 |
| 40 | sp\|Q9Y2H1\|ST38L_HUMAN | 142 | 54196 | 0.6 |
| 41 | sp\|P13647\|K2C5_HUMAN | 143 | 62568 | 0.59 |
| 42 | sp\|P05783\|K1C18_HUMAN | 129 | 48029 | 0.59 |
| 43 | sp\|Q70IA6\|MOB2_HUMAN | 89 | 27251 | 0.59 |
| 44 | sp\|Q5VTE0\|EF1A3_HUMAN | 141 | 50495 | 0.56 |
| 45 | sp\|O14744\|ANM5_HUMAN | 108 | 73322 | 0.55 |
| 46 | sp\|P10412\|H14_HUMAN | 116 | 21852 | 0.53 |
| 47 | sp\|P62861\|RS30_HUMAN | 53 | 14438 | 0.53 |
| 48 | sp\|P53999\|TCP4_HUMAN | 34 | 14386 | 0.53 |
| 49 | sp\|P35527\|K1C9_HUMAN | 339 | 62255 | 0.51 |
| 50 | sp\|Q5TEC6\|H37_HUMAN | 45 | 15478 | 0.49 |
| 51 | sp\|P15880\|RS2_HUMAN | 32 | 31590 | 0.49 |
| 52 | sp\|P62826\|RAN_HUMAN | 72 | 24579 | 0.47 |
| 53 | sp\|P62266\|RS23_HUMAN | 62 | 15969 | 0.47 |
| 54 | sp\|Q9BQE3\|TBA1C_HUMAN | 89 | 50548 | 0.46 |
| 55 | sp\|O60869\|EDF1_HUMAN | 31 | 16359 | 0.46 |
| 56 | sp\|P46776\|RL27A_HUMAN | 60 | 16665 | 0.45 |
| 57 | sp\|O14602\|IF1AY_HUMAN | 32 | 16546 | 0.45 |
| 58 | sp\|P19474\|RO52_HUMAN | 91 | 55162 | 0.42 |
| 59 | sp\|P60842\|IF4A1_HUMAN | 117 | 46353 | 0.41 |
| 60 | sp\|P62917\|RL8_HUMAN | 48 | 28235 | 0.4 |
| 61 | sp\|P62280\|RS11_HUMAN | 38 | 18590 | 0.4 |
| 62 | sp\|P14618\|KPYM_HUMAN | 96 | 58470 | 0.39 |
| 63 | sp\|P46778\|RL21_HUMAN | 33 | 18610 | 0.39 |
| 64 | sp\|P25705\|ATPA_HUMAN | 115 | 59828 | 0.38 |
| 65 | sp\|P07437\|TBB5_HUMAN | 93 | 50095 | 0.38 |
| 66 | sp\|Q13509\|TBB3_HUMAN | 75 | 50856 | 0.37 |
| 67 | sp\|Q6P3W7\|SCYL2_HUMAN | 139 | 104327 | 0.36 |
| 68 | sp\|P09038\|FGF2_HUMAN | 111 | 31093 | 0.36 |
| 69 | sp\|Q02543\|RL18A_HUMAN | 50 | 21034 | 0.35 |
| 70 | sp\|P06576\|ATPB_HUMAN | 90 | 56525 | 0.33 |
| 71 | sp\|P06753\|TPM3_HUMAN | 54 | 32987 | 0.33 |
| 72 | sp\|P32969\|RL9_HUMAN | 26 | 21964 | 0.33 |
| 73 | sp\|P46781\|RS9_HUMAN | 59 | 22635 | 0.32 |
| 74 | sp\|P05109\|S10A8_HUMAN | 27 | 10885 | 0.32 |
| 75 | sp\|P11021\|BIP_HUMAN | 178 | 72402 | 0.3 |
| 76 | sp\|P04406\|G3P_HUMAN | 70 | 36201 | 0.3 |
| 77 | sp\|P81605\|DCD_HUMAN | 29 | 11391 | 0.3 |
| 78 | sp\|P26373\|RL13_HUMAN | 52 | 24304 | 0.29 |
| 79 | sp\|P00338\|LDHA_HUMAN | 43 | 36950 | 0.29 |
| 80 | sp\|Q00839\|HNRPU_HUMAN | 179 | 91269 | 0.28 |
| 81 | sp\|Q6NVV1\|R13P3_HUMAN | 48 | 12184 | 0.28 |
| 82 | sp\|P28072\|PSB6_HUMAN | 38 | 25570 | 0.28 |
| 83 | sp\|P23588\|IF4B_HUMAN | 100 | 69167 | 0.26 |
| 84 | sp\|P60866\|RS20_HUMAN | 60 | 13478 | 0.25 |
| 85 | sp\|P0C0S5\|H2AZ_HUMAN | 33 | 13545 | 0.25 |
| 86 | sp\|P62701\|RS4X_HUMAN | 72 | 29807 | 0.24 |
| 87 | sp\|P62899\|RL31_HUMAN | 70 | 14454 | 0.24 |
| 88 | sp\|Q9Y657\|SPIN1_HUMAN | 58 | 29696 | 0.24 |
| 89 | sp\|P62753\|RS6_HUMAN | 49 | 28834 | 0.24 |
| 90 | sp\|P62424\|RL7A_HUMAN | 30 | 30148 | 0.23 |
| 91 | sp\|P35580\|MYH10_HUMAN | 278 | 229827 | 0.22 |
| 92 | sp\|P36578\|RL4_HUMAN | 70 | 47953 | 0.22 |
| 93 | sp\|P46779\|RL28_HUMAN | 30 | 15795 | 0.22 |
| 94 | sp\|P08238\|HS90B_HUMAN | 106 | 83554 | 0.21 |
| 95 | sp\|Q3ZCM7\|TBB8_HUMAN | 87 | 50257 | 0.21 |
| 96 | sp\|Q99623\|PHB2_HUMAN | 71 | 33276 | 0.21 |
| 97 | sp\|P02042\|HBD_HUMAN | 28 | 16159 | 0.21 |
| 98 | sp\|P02768\|ALBU_HUMAN | 139 | 71317 | 0.2 |
| 99 | sp\|P35637\|FUS_HUMAN | 68 | 53622 | 0.2 |
| 100 | sp\|P61626\|LYSC_HUMAN | 51 | 16982 | 0.2 |
| 101 | sp\|P40763\|STAT3_HUMAN | 44 | 88810 | 0.2 |
| 102 | sp\|P98179\|RBM3_HUMAN | 22 | 17160 | 0.2 |
| 103 | sp\|Q03393\|PTPS_HUMAN | 18 | 16489 | 0.2 |
| 104 | sp\|P02545\|LMNA_HUMAN | 92 | 74380 | 0.19 |
| 105 | sp\|P47914\|RL29_HUMAN | 72 | 17798 | 0.19 |
| 106 | sp\|P09651\|ROA1_HUMAN | 104 | 38837 | 0.18 |
| 107 | sp\|P62979\|RS27A_HUMAN | 49 | 18296 | 0.18 |
| 108 | sp\|Q14011\|CIRBP_HUMAN | 48 | 18637 | 0.18 |
| 109 | sp\|P07900\|HS90A_HUMAN | 67 | 85006 | 0.16 |
| 110 | sp\|P62913\|RL11_HUMAN | 41 | 20468 | 0.16 |
| 111 | sp\|P18085\|ARF4_HUMAN | 37 | 20612 | 0.16 |
| 112 | sp\|Q9NXS2\|QPCTL_HUMAN | 34 | 43068 | 0.16 |
| 113 | sp\|P18621\|RL17_HUMAN | 30 | 21611 | 0.16 |
| 114 | sp\|Q96T59\|CDRTF_HUMAN | 23 | 20980 | 0.16 |
| 115 | sp\|P39023\|RL3_HUMAN | 79 | 46365 | 0.15 |
| 116 | sp\|P0DMV8\|HS71A_HUMAN | 72 | 70294 | 0.15 |
| 117 | sp\|P04792\|HSPB1_HUMAN | 33 | 22826 | 0.15 |
| 118 | sp\|Q96LR5\|UB2E2_HUMAN | 30 | 22526 | 0.15 |
| 119 | sp\|Q14183\|DOC2A_HUMAN | 19 | 44502 | 0.15 |
| 120 | sp\|P26641\|EF1G_HUMAN | 76 | 50429 | 0.14 |
| 121 | sp\|P50914\|RL14_HUMAN | 64 | 23531 | 0.14 |
| 122 | sp\|O00571\|DDX3X_HUMAN | 52 | 73597 | 0.14 |
| 123 | sp\|P62241\|RS8_HUMAN | 35 | 24475 | 0.14 |
| 124 | sp\|P61313\|RL15_HUMAN | 33 | 24245 | 0.14 |
| 125 | sp\|P24534\|EF1B_HUMAN | 60 | 24919 | 0.13 |
| 126 | sp\|Q96CT7\|CC124_HUMAN | 48 | 25820 | 0.13 |
| 127 | sp\|P30041\|PRDX6_HUMAN | 41 | 25133 | 0.13 |
| 128 | sp\|Q00688\|FKBP3_HUMAN | 34 | 25218 | 0.13 |
| 129 | sp\|Q6IQ22\|RAB12_HUMAN | 49 | 27573 | 0.12 |
| 130 | sp\|Q86V81\|THOC4_HUMAN | 39 | 26872 | 0.12 |
| 131 | sp\|Q9BUZ4\|TRAF4_HUMAN | 33 | 55218 | 0.12 |
| 132 | sp\|Q8TAA3\|PSMA8_HUMAN | 33 | 28683 | 0.12 |
| 133 | sp\|Q01081\|U2AF1_HUMAN | 33 | 28368 | 0.12 |
| 134 | sp\|P17483\|HXB4_HUMAN | 22 | 27986 | 0.12 |
| 135 | sp\|P15559\|NQO1_HUMAN | 42 | 30905 | 0.11 |
| 136 | sp\|P10809\|CH60_HUMAN | 41 | 61187 | 0.11 |
| 137 | sp\|Q00059\|TFAM_HUMAN | 41 | 29306 | 0.11 |
| 138 | sp\|P25789\|PSA4_HUMAN | 36 | 29750 | 0.11 |
| 139 | sp\|Q14331\|FRG1_HUMAN | 36 | 29439 | 0.11 |
| 140 | sp\|P29692\|EF1D_HUMAN | 34 | 31217 | 0.11 |
| 141 | sp\|P16152\|CBR1_HUMAN | 34 | 30641 | 0.11 |
| 142 | sp\|Q9BU76\|MMTA2_HUMAN | 33 | 29679 | 0.11 |
| 143 | sp\|P47756\|CAPZB_HUMAN | 32 | 31616 | 0.11 |
| 144 | sp\|P17987\|TCPA_HUMAN | 31 | 60819 | 0.11 |
| 145 | sp\|P47755\|CAZA2_HUMAN | 55 | 33157 | 0.1 |
| 146 | sp\|Q9NWB6\|ARGL1_HUMAN | 44 | 33197 | 0.1 |
| 147 | sp\|Q96HS1\|PGAM5_HUMAN | 40 | 32213 | 0.1 |
| 148 | sp\|P45880\|VDAC2_HUMAN | 39 | 32060 | 0.1 |
| 149 | sp\|P46777\|RL5_HUMAN | 33 | 34569 | 0.1 |
| 150 | sp\|A6NHQ2\|FBLL1_HUMAN | 28 | 34839 | 0.1 |
| 151 | sp\|Q96A73\|P33MX_HUMAN | 26 | 33226 | 0.1 |
| 152 | sp\|P23246\|SFPQ_HUMAN | 41 | 76216 | 0.09 |
| 153 | sp\|P01859\|IGHG2_HUMAN | 40 | 36505 | 0.09 |
| 154 | sp\|Q9NZ01\|TECR_HUMAN | 40 | 36410 | 0.09 |
| 155 | sp\|P63244\|RACK1_HUMAN | 37 | 35511 | 0.09 |
| 156 | sp\|Q9H9B4\|SFXN1_HUMAN | 32 | 35881 | 0.09 |
| 157 | sp\|Q96T54\|KCNKH_HUMAN | 29 | 37327 | 0.09 |
| 158 | sp\|O00303\|EIF3F_HUMAN | 27 | 37654 | 0.09 |
| 159 | sp\|Q13347\|EIF3I_HUMAN | 23 | 36878 | 0.09 |
| 160 | sp\|O15397\|IPO8_HUMAN | 59 | 120945 | 0.08 |
| 161 | sp\|Q9BUA3\|SPNDC_HUMAN | 49 | 41297 | 0.08 |
| 162 | sp\|Q12931\|TRAP1_HUMAN | 40 | 80345 | 0.08 |
| 163 | sp\|Q3ZCQ8\|TIM50_HUMAN | 32 | 39850 | 0.08 |
| 164 | sp\|P04075\|ALDOA_HUMAN | 31 | 39851 | 0.08 |
| 165 | sp\|P33993\|MCM7_HUMAN | 31 | 81884 | 0.08 |
| 166 | sp\|P06733\|ENOA_HUMAN | 84 | 47481 | 0.07 |
| 167 | sp\|P50454\|SERPH_HUMAN | 57 | 46525 | 0.07 |
| 168 | sp\|P31943\|HNRH1_HUMAN | 56 | 49484 | 0.07 |
| 169 | sp\|P13639\|EF2_HUMAN | 54 | 96246 | 0.07 |
| 170 | sp\|P14625\|ENPL_HUMAN | 54 | 92696 | 0.07 |
| 171 | sp\|Q96CW1\|AP2M1_HUMAN | 41 | 49965 | 0.07 |
| 172 | sp\|O00148\|DX39A_HUMAN | 39 | 49611 | 0.07 |
| 173 | sp\|P23526\|SAHH_HUMAN | 36 | 48255 | 0.07 |
| 174 | sp\|P62495\|ERF1_HUMAN | 29 | 49228 | 0.07 |
| 175 | sp\|Q9Y6N5\|SQOR_HUMAN | 27 | 50214 | 0.07 |
| 176 | sp\|P49411\|EFTU_HUMAN | 24 | 49852 | 0.07 |
| 177 | sp\|Q96GA3\|LTV1_HUMAN | 47 | 55049 | 0.06 |
| 178 | sp\|P55084\|ECHB_HUMAN | 45 | 51547 | 0.06 |
| 179 | sp\|B5ME19\|EIFCL_HUMAN | 44 | 106091 | 0.06 |
| 180 | sp\|P23381\|SYWC_HUMAN | 41 | 53474 | 0.06 |
| 181 | sp\|O60701\|UGDH_HUMAN | 40 | 55674 | 0.06 |
| 182 | sp\|Q8NCA5\|FA98A_HUMAN | 40 | 55694 | 0.06 |
| 183 | sp\|P39656\|OST48_HUMAN | 38 | 50940 | 0.06 |
| 184 | sp\|Q9NRH3\|TBG2_HUMAN | 33 | 51402 | 0.06 |
| 185 | sp\|P40227\|TCPZ_HUMAN | 32 | 58444 | 0.06 |
| 186 | sp\|P09914\|IFIT1_HUMAN | 31 | 55781 | 0.06 |
| 187 | sp\|P30101\|PDIA3_HUMAN | 31 | 57146 | 0.06 |
| 188 | sp\|Q9Y230\|RUVB2_HUMAN | 30 | 51296 | 0.06 |
| 189 | sp\|Q15233\|NONO_HUMAN | 29 | 54311 | 0.06 |
| 190 | sp\|P43490\|NAMPT_HUMAN | 28 | 55772 | 0.06 |
| 191 | sp\|Q96IF1\|AJUBA_HUMAN | 26 | 58722 | 0.06 |
| 192 | sp\|Q86YJ6\|THNS2_HUMAN | 18 | 54652 | 0.06 |
| 193 | sp\|Q9H3Z7\|ABHGB_HUMAN | 17 | 53205 | 0.06 |
| 194 | sp\|P49327\|FAS_HUMAN | 66 | 275877 | 0.05 |
| 195 | sp\|P50990\|TCPQ_HUMAN | 54 | 60153 | 0.05 |
| 196 | sp\|Q01844\|EWS_HUMAN | 45 | 68721 | 0.05 |
| 197 | sp\|P49368\|TCPG_HUMAN | 44 | 61066 | 0.05 |
| 198 | sp\|Q9H361\|PABP3_HUMAN | 34 | 70215 | 0.05 |
| 199 | sp\|Q16875\|F263_HUMAN | 33 | 60370 | 0.05 |
| 200 | sp\|Q5JTV8\|TOIP1_HUMAN | 31 | 66379 | 0.05 |
| 201 | sp\|P08195\|4F2_HUMAN | 27 | 68180 | 0.05 |
| 202 | sp\|P17844\|DDX5_HUMAN | 25 | 69618 | 0.05 |
| 203 | sp\|Q96N38\|ZN714_HUMAN | 20 | 65837 | 0.05 |
| 204 | sp\|Q5VW38\|GP107_HUMAN | 14 | 67290 | 0.05 |
| 205 | sp\|P38646\|GRP75_HUMAN | 46 | 73920 | 0.04 |
| 206 | sp\|Q92499\|DDX1_HUMAN | 38 | 83349 | 0.04 |
| 207 | sp\|Q9ULK4\|MED23_HUMAN | 36 | 158254 | 0.04 |
| 208 | sp\|O60331\|PI51C_HUMAN | 36 | 73500 | 0.04 |
| 209 | sp\|O00469\|PLOD2_HUMAN | 34 | 85373 | 0.04 |
| 210 | sp\|Q8TF76\|HASP_HUMAN | 32 | 89637 | 0.04 |
| 211 | sp\|Q08J23\|NSUN2_HUMAN | 27 | 87214 | 0.04 |
| 212 | sp\|Q2PZI1\|D19L1_HUMAN | 26 | 78180 | 0.04 |
| 213 | sp\|P11387\|TOP1_HUMAN | 25 | 91125 | 0.04 |
| 214 | sp\|O14639\|ABLM1_HUMAN | 24 | 89513 | 0.04 |
| 215 | sp\|Q06210\|GFPT1_HUMAN | 24 | 79555 | 0.04 |
| 216 | sp\|Q96G46\|DUS3L_HUMAN | 23 | 73688 | 0.04 |
| 217 | sp\|Q08043\|ACTN3_HUMAN | 44 | 103917 | 0.03 |
| 218 | sp\|Q13200\|PSMD2_HUMAN | 44 | 100877 | 0.03 |
| 219 | sp\|O00268\|TAF4_HUMAN | 37 | 110332 | 0.03 |
| 220 | sp\|Q9Y2W1\|TR150_HUMAN | 35 | 108658 | 0.03 |
| 221 | sp\|Q7Z2W4\|ZCCHV_HUMAN | 32 | 103135 | 0.03 |
| 222 | sp\|P98175\|RBM10_HUMAN | 29 | 103811 | 0.03 |
| 223 | sp\|O75164\|KDM4A_HUMAN | 23 | 122581 | 0.03 |
| 224 | sp\|Q9NXL6\|SIDT1_HUMAN | 23 | 94747 | 0.03 |
| 225 | sp\|Q86Z14\|KLOTB_HUMAN | 22 | 120473 | 0.03 |
| 226 | sp\|P41252\|SYIC_HUMAN | 49 | 145718 | 0.02 |
| 227 | sp\|P31327\|CPSM_HUMAN | 45 | 165975 | 0.02 |
| 228 | sp\|O75882\|ATRN_HUMAN | 31 | 163450 | 0.02 |
| 229 | sp\|Q9Y2K9\|STB5L_HUMAN | 30 | 133570 | 0.02 |
| 230 | sp\|P08575\|PTPRC_HUMAN | 29 | 148876 | 0.02 |
| 231 | sp\|Q9NYU2\|UGGG1_HUMAN | 24 | 177819 | 0.02 |
| 232 | sp\|Q14152\|EIF3A_HUMAN | 23 | 166867 | 0.02 |
| 233 | sp\|Q86VP6\|CAND1_HUMAN | 19 | 137999 | 0.02 |
| 234 | sp\|O60318\|GANP_HUMAN | 32 | 220662 | 0.01 |
| 235 | sp\|Q15772\|SPEG_HUMAN | 28 | 356865 | 0.01 |
| 236 | sp\|Q8N1I0\|DOCK4_HUMAN | 28 | 226888 | 0.01 |
| 237 | sp\|Q15149\|PLEC_HUMAN | 26 | 533462 | 0.01 |
| 238 | sp\|Q5JSL3\|DOC11_HUMAN | 25 | 240142 | 0.01 |
| 239 | sp\|Q8NEV8\|EXPH5_HUMAN | 17 | 223752 | 0.01 |
| 240 | sp\|Q8NF91\|SYNE1_HUMAN | 36 | 1017127 | 0 |

**Table S4. Correlation between circMAST1 expression and clinicopathologic characteristics of CCa**

| Characteristics | Total | circMAST1 expression | | *P* value |
| --- | --- | --- | --- | --- |
|  | 131 | High | Low |  |
| Age (years) |  |  |  | 0.7966 |
| < 42 | 32 | 17 | 15 |  |
| ≥ 42 | 99 | 50 | 49 |  |
| FIGO stage |  |  |  | 0.6146 |
| Ⅰ (Ⅰa2 + Ⅰb1 + Ⅰb2) | 83 | 47 | 36 |  |
| Ⅱ (Ⅱa1 + Ⅱa2) | 48 | 25 | 23 |  |
| Tumor size (cm) |  |  |  | 0.0004 *** |
| ≤ 4 | 100 | 59 | 41 |  |
| > 4 | 31 | 7 | 24 |  |
| Pathologic types |  |  |  | 0.6723 |
| Squamous cell carcinoma | 103 | 56 | 47 |  |
| Adenocarcinoma | 18 | 10 | 8 |  |
| [Adenosquamous](../../../../../../è½¯ä»¶/Youdao/Dict/8.7.0.0/resultui/html/index.html#/javascript:;) [carcinoma](../../../../../../è½¯ä»¶/Youdao/Dict/8.7.0.0/resultui/html/index.html#/javascript:;) | 10 | 4 | 6 |  |
| Differentiation |  |  |  | 0.6945 |
| Well | 19 | 11 | 8 |  |
| Moderate | 42 | 20 | 22 |  |
| Poor | 70 | 33 | 37 |  |
| Stromal invasion |  |  |  | 0.3483 |
| < 1/2 | 93 | 50 | 43 |  |
| ≥ 1/2 | 38 | 17 | 21 |  |
| LVSI |  |  |  | 0.6191 |
| Positive | 43 | 23 | 20 |  |
| Negative | 88 | 43 | 45 |  |
| LNM |  |  |  | 0.0169* |
| Positive | 35 | 10 | 25 |  |
| Negative | 96 | 50 | 46 |  |
| Vaginal invasion |  |  |  | 0.4790 |
| Positive | 11 | 4 | 7 |  |
| Negative | 122 | 64 | 58 |  |
| Parametrial invasion |  |  |  | 0.7471 |
| Positive | 6 | 3 | 3 |  |
| Negative | 125 | 65 | 60 |  |

χ^2^-test. FIGO: the International Federation of Gynecology and Obstetrics; LVSI, lymphovascular space invasion; LNM, lymph node metastasis.
